# What is the optimal dose of adipose-derived mesenchymal stem cells treatment for knee osteoarthritis? A conventional and network meta-analysis of randomized controlled trials

**DOI:** 10.1186/s13287-023-03475-5

**Published:** 2023-09-12

**Authors:** Zongyuan Huang, Shuai Zhang, Mingde Cao, Zhujian Lin, Ling Kong, Xin Wu, Qianshi Guo, Yuxiang Ouyang, Yancheng Song

**Affiliations:** 1 Department of Orthopedics, The First Affiliated Hospital of Guangdong Pharmaceutical University, Guangdong Pharmaceutical University, No. 19 Nonglinxia Road, Yuexiu District, Guangzhou, Guangdong Province China; 2 Department of Orthopaedics and Traumatology, Faculty of Medicine, The Chinese University of Hong Kong, Hong Kong, China; 3 Guangzhou Regenerative Medicine and Health Guangdong Laboratory, Guangzhou, China

**Keywords:** Mesenchymal stem cells, Cell doses, Knee osteoarthritis, Meta-analysis

## Abstract

**Background:**

Despite increasing clinical investigations underscoring the efficacy and safety of adipose-derived mesenchymal stem cells (AD-MSCs) therapy in knee osteoarthritis (KOA), no article has recently reviewed the cell dosage. This study aimed to evaluate the efficacy and safety of varying doses of AD-MSCs in treating KOA using conventional and network meta-analysis.

**Methods:**

A search of databases in in Chinese and English was performed to identify randomized controlled trials (RCT) on MSCs for knee osteoarthritis from the inception date to May 1, 2022. This study mainly analyzed the efficacy of AD-MSCs in the treatment of KOA, and subgroup analysis was performed on the therapeutic effects of MSCs from different tissues at the same dose. We divided the different cell doses into low, moderate, and high groups, with the corresponding cell doses: (0–25)*10^6, (25–50)*10^6, and > 50*10^6 cells, respectively. We further analyzed the improvement of improvement of the Visual Analog Scale (VAS) and the Western Ontario and McMaster Universities Arthritis Index (WOMAC) scores and the incidence of adverse events (AEs) after varied dosage injection.

**Results:**

A total of 16 literatures were included in this study, of which 8 literatures were about AD-MSCs. Conventional meta-analysis suggests that AD-MSCs can reduce pain and improve function in KOA patients, regardless of the cell doses, up to 12 months of follow-up. The network meta-analysis showed that intra-articular injection of AD-MSCs significantly improved pain and knee function scores in KOA patients compared with the control group at 3, 6, and 12 months. Among the three groups, the high-dose group had the best treatment effect, and the degree of joint pain and dysfunction indicators improved more significantly in the early stage. For adverse events, there was a dose–response trend that increased with increasing doses.

**Conclusions:**

Both cell doses reduced pain and improved knee function in KOA patients. The effect surpassed in the high-dose group than in the moderate-dose, low-dose and control groups. However, adverse events also increase with the increase in dose, which should be carefully considered in clinical application, and the side effects still need to be paid attention to. Considering the limitations of this meta-analysis, future studies need to further explore the efficacy and safety of different doses of treatment, and carry out large sample, multi-center, randomized controlled trials to ensure the reliability and promotion value of the research results.

## Introduction

Knee osteoarthritis (KOA) is a chronic disabling condition common in middle-aged and older people, characterized by symptoms such as pain, knee joint swelling, and restricted mobility [1]. Studies have shown that in adults aged 60 years or older, the prevalence of KOA is approximately 10% in males and 13% in females, with over 250 million individuals worldwide being affected by this condition [2]. Moreover, younger demographics are showing an escalation in the disease's incidence [3]. At present, the clinical treatment of OA mainly includes conservative treatments and surgical interventions. Conservative treatments include anti-inflammatory analgesics, intra-articular injections of hormone or sodium hyaluronate, rehabilitation physiotherapy, etc., which can relieve pain and mitigate symptoms. However, the benefits are temporary, and it is difficult to prevent or control the OA progression [4, 5], so that the disease is progressively aggravated, and finally surgical treatment is necessary. Surgical treatment is a palliative intervention for end-stage OA, which is not suitable for young patients and the elderly who cannot tolerate surgery. In addition, the life of artificial joints is limited, and there are many postoperative complications. Therefore, the prominent imperative is to develop effective treatments that simultaneously manage common symptoms and slow down KOA progression.

In recent years, intra-articular injection of mesenchymal stem cells (MSCs) has received extensive attention due to its immunomodulatory potential, offering a promising avenue toward decelerating the KOA development. MSCs derive from various sources. At present, the common MSCs that have been used in clinical trials of KOA include Adipose-derived mesenchymal stem cells (AD-MSCs), Bone marrow mesenchymal stem cells (BM-MSCs), and Umbilical cord mesenchymal stem cells (UC-MSCs). Clinical trials showed favored results [6–9].

However, there are no standards for the dose of MSCs used in clinical trials. Different research teams using the same cell source vary the number of cells from 10^6^ to 10^8^, and the optimal dose remains unknown. Therefore, we need to establish an accurate, effective, and standardized treatment protocol to choose different MSC sources of MSCs and their injection doses. The differences in biological functions such as surface markers, immunophenotype, migration, proliferation, and differentiation of MSCs from different tissues may affect the therapeutic effect. Therefore, this study mainly focused on AD-MSCs, which have the most abundant tissue sources and are the most widely used. In addition, we divided different doses of AD-MSCs into three groups, high, moderate, and low, to verify the effectiveness of different doses in the treatment of KOA by conventional meta-analysis. At the same time, due to the lack of direct comparative evidence on different doses of MSCs, their effects were indirectly compared using network meta-analysis to provide evidence for the optimal cell dose for current KOA treatment.

## Methods and materials

This article mainly analyzes the efficacy of different doses of AD-MSCs in the treatment of KOA. In order to conduct a subgroup analysis of the therapeutic effect of MSCs from different tissues under the same dose, the literature related to UC-MSCs and BM-MSCs was included. This systematic review was completed following the Preferred Reporting Items for Systematic Reviews and Meta-Analysis (PRISMA) statement [10]. A meta-analysis of published literature was performed, which did not require the approval of an ethics committee. In addition, the meta-analysis protocol has been submitted to PROSPERO under registration number CRD42022378457.

### Search results

We systematically searched PubMed, EMBASE, Web of Science, The Cochrane Library, CNKI, Wan Fang, and VIP for relevant studies from inception to May 1, 2022, limited to papers related to the treatment of KOA by MSCs via joint injection. In addition, for a comprehensive search, all relevant articles in English and Chinese were searched, and relevant randomized controlled trials (RCTs) were retrieved from clinical trial reports or review references.

The following search terms were used: Osteoarthritis, osteoarthritides, osteoarthrosis, osteoarthroses, osteoarthritic, degenerative arthritides, degenerative arthritis, osteoarthrosis deformans, arthrosis, arthroses, mesenchymal stem cells, mesenchymal stem cell, stem cell, stem cells, stromal cell, stromal cells, progenitor cell, progenitor cells, Mesenchymal, Knee, knees, randomized controlled trial, controlled clinical trial. The complete search strategy is provided in Appendix 1.

### Inclusion and exclusion criteria

The inclusion and exclusion process followed the PICOS (Participants, Intervention, Comparison, Outcome, and Study) principle. (1) Participants: adult patients diagnosed with primary knee osteoarthritis according to the guidelines formulated by the American Rheumatology Society or the Chinese Orthopaedic Association. (2) Intervention: the experimental group only received a single injection of MSCs into the knee joint. Those who received multiple injections or other routes of administration were excluded. The cell doses were divided into low, moderate, and high groups, and the corresponding cell doses were as follows: (0 < cells ≤ 25)*10^6, (25 < cells ≤ 50)*10^6, and cells > 50*10^6 respectively. (3) Comparison: The control group was treated with intra-articular injection of either normal saline, hormone, sodium hyaluronate, and cell culture medium and was represented as "Standard". (4) Outcome: at least one of Visual Analog Scale (VAS), Western Ontario and McMaster Universities Osteoarthritis Index (WOMAC), and Adverse Events (AEs) were included in the article. (5) Study: Chinese or English randomized controlled trials without the restriction of publication year. (6) Exclusion criteria included: a. Patients with intra-segmental fractures or cruciate ligament injuries; b. Articles of poor quality, duplicate publication, and incomplete data; c. Reviews, case reports, conference papers, and animal experiments.

### Literature screening and data extraction

To ensure the accuracy and reliability of data extraction, two researchers independently read the titles and abstracts and excluded articles that did not meet the inclusion criteria. The full text was then read to screen out eligible articles further. A unified data extraction table was designed. The extracted information mainly included title, first author, publication year, country, sample size, mean age, intervention methods, follow-up period, and outcome indicators. In case of disagreement, the decision was discussed with a third researcher. All continuous variables are expressed as mean ± SD. If the mean value or standard deviation of the study results were not provided, median, IQR, SE, *P* value, and 95% CI value in the Cochrane handbook were used to calculate the mean and standard deviation [11].

### Quality assessment

Two investigators independently evaluated the literature and performed a preliminary screening according to the inclusion and exclusion criteria. The third researcher judged the literature with dissent, resolved the differences raised by the earlier researchers, and finally discussed and reached a consensus. The quality of the final included literature was assessed using the Cochrane tool for assessing the risk of bias [11]. The evaluation included the following seven aspects: ① random sequence generation; ② allocation concealment; ③ blinding of participants and personnel; ④ blinding of outcome assessment; ⑤ incomplete outcome data; ⑥ selective reporting and other bias. These points were graded as low risk, unclear, and high risk.

### Primary outcome

The primary outcomes include ① WOMAC of 3, 6, and 12 months; ② VAS of 3, 6, and 12 months; ③ Occurrence of adverse events. Based on the different control groups used in various kinds of literature, the WOMAC and VAS scores before treatment in the experimental group were used as the common control group to reduce the source of heterogeneity.

### Statistical analysis

Conventional meta-analysis was performed using Review Manager 5.4 to verify the efficacy of three doses of AD-MACs in treating KOA. The VAS scores of MSCs derived from different tissues at the same intervention dose were analyzed in subgroups. Chi-squared test was used to evaluate the statistical heterogeneity among the studies. *I*^2^ was used to assess literature heterogeneity; *I*^2^ > 50% suggested high heterogeneity, and the random effects model was used, and when *I*^2^ ≤ 50% indicated lesser heterogeneity, the fixed effects model was used. Standardized mean difference (SMD) and 95% credible intervals (CI) were used for continuous variables. No statistical significance was considered if 0 was included in the 95% confidence interval. Funnel plots were used to assess publication bias.

Network Meta-analysis: Frequency network meta-analysis was performed in Stata 16.0 software to analyze the differences in the efficacy of three different cell doses. The surface area under the cumulative ranking curves (SUCRA) was used to rank the results of interventions. A SUCRA score closer to 100% indicates a better therapeutic effect of the intervention. *P* < 0.05 was considered significant. A funnel plot was made for the outcome indicators to evaluate publication bias and small sample effect.

## Results

### Results of the search

**Fig. 1:**
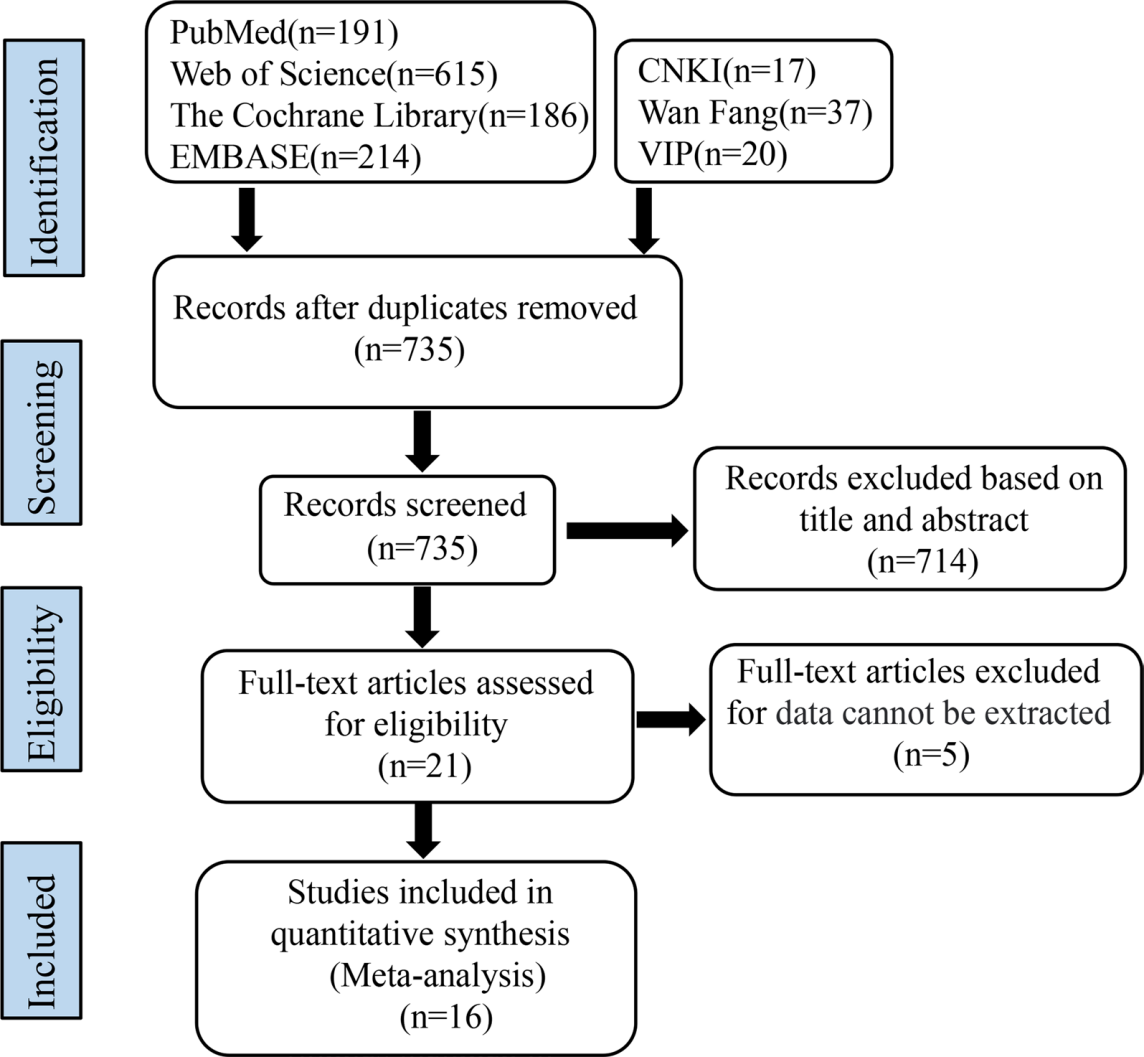
Flowchart depicting the workflow of literature screening

A preliminary search identified 1280 relevant articles for this study. After thoroughly screening each layer, 16 randomized controlled trials were finally selected, including 14 in English and 2 in Chinese. Among them, 8 articles related to AD-MSCs, 5 articles related to BM-MSCs, and 3 articles related to UC-MSCs. The flowchart of the method followed for the literature screening is shown in Fig. 1.

### Description of the trials included in this study

We included 16 RCTs involving 659 participants for this study. The injection doses ranged from 3.9 × 10^6^ to 150 × 10^6^ cells. Some RCTs included two or more experimental groups, with the control group divided into two or more equal parts to match the experimental groups. The matched groups have been labeled as (1) and (2). The study characteristics are shown in Table 1.

**Table 1:** Basic characteristics of the literature included in the study

References	Study	Country	Origin	Cell doses	Kellgren–Lawrence grade	Sample size (*M*/*F*)		Mean age (years)		Follow-up (month)	Relevant outcomes
						Trial group	Control group	Trial group	Control group		
[12]	Freitag et al., (1) 2019	Australia	AD-MSCs	100 × 10^6^	II,III	10 (7/3)	10 (5/5)	54.60 ± 8.10	51.10 ± 6.10	1, 3, 6, 12	①③④⑦
	Freitag et al., (2) 2019	Australia	AD-MSCs	100 × 10^6^	II,III	10 (4/6)	10 (5/5)	54.60 ± 8.10	51.10 ± 6.10	1, 3, 6, 12	①③④⑦
[13]	Garza et al., (1) 2020	USA	AD-MSCs	15 × 10^6^	II,III	13 (4/9)	13 (6/7)	60.50 ± 7.90	57.10 ± 9.10	6, 12	①④⑦
	Garza et al., (2) 2020	USA	AD-MSCs	30 × 10^6^	II,III	13 (6/7)	13 (6/7)	59.50 ± 11.70	57.10 ± 9.10	6, 12	①④⑦
[14]	Koh et al., 2012	South Korea	AD-MSCs	1.89 × 10^6^	II,III, IV	25 (8/17)	25 (8/17)	54.20 ± 9.30	54.40 ± 11.30	3, 12	②⑧⑦
[15]	Lee et al., 2019	South Korea	AD-MSCs	100 × 10^6^	II,III, IV	12 (3/9)	12 (3/9)	62.20 ± 6.50	63.20 ± 4.20	1, 3, 6	①②③④⑥⑦
[16]	Lu et al., 2019	China	AD-MSCs	50 × 10^6^	I,II,III	26 (3/23)	26 (3/23)	55.03 ± 9.19	55.03 ± 9.19	6, 12	①②④⑥⑦
[17]	Jo et al., (1) 2017	Korea	AD-MSCs	10 × 10^6^	III, IV	3	–	61.80 ± 6.60	–	1, 2, 3, 6, 12, 24	①②③④
	Jo et al., (2) 2017	Korea	AD-MSCs	50 × 10^6^	III, IV	3	–	61.80 ± 6.60	–	1, 2, 3, 6, 12, 24	①②③④
	Jo et al., (3) 2017	Korea	AD-MSCs	100 × 10^6^	III, IV	12	–	61.80 ± 6.60	–	1, 2, 3, 6, 12, 24	①②③④
[18]	Kuah et al., (1) 2018	Australia	AD-MSCs	3.9 × 10^6^	I,II,III	8 (8/2)	4 (1/3)	52.90 ± 6.47	55.00 ± 10.42	1, 3, 6, 9, 12	①②④
	Kuah et al., (1) 2018	Australia	AD-MSCs	6.7 × 10^6^	I,II,III	8 (5/3)	4 (1/3)	52.90 ± 6.47	55.00 ± 10.42	1, 3, 6, 9, 12	①②④
[19]	Chen et al., (1) 2021	China	AD-MSCs	16 × 10^6^	I,II,III	17 (3/17)	8 (3/5)	67.70 ± 6.84	70.50 ± 8.37	1, 3, 6, 12, 24	①②
	Chen et al., (2) 2021	China	AD-MSCs	32 × 10^6^	I,II,III	17 (2/15)	8 (3/5)	68.60 ± 6.45	70.50 ± 8.37	1, 3, 6, 12, 24	①②
	Chen et al., (3) 2021	China	AD-MSCs	64 × 10^6^	I,II,III	15 (3/12)	8 (3/5)	64.90 ± 4.91	70.50 ± 8.37	1, 3, 6, 12, 24	①②
[20]	Bastos et al., 2019	Portugal	BM-MSCs	40 × 10^6^	I,II,III,IV	16 (10/6)	17 (9/8)	55.70 ± 7.8	55.90 ± 13.4	1, 2, 3, 6, 9, 12	⑦
[21]	Lamo‑Espinos et al., (1) 2016	Spain	BM-MSCs	10 × 10^6^	II,III,IV	10 (4/6)	10 (7/3)	65.90 ± 8.22	60.30 ± 4.44	3, 6, 12	①②④
	Lamo‑Espinos et al., (1) 2016	Spain	BM-MSCs	100 × 10^6^	II,III,IV	10 (8/2)	10 (7/3)	57.80 ± 4.29	60.30 ± 4.44	3, 6, 12	①②④
[22]	Emadedin et al., 2018	Iran	BM-MSCs	40 × 10^6^	II,III,IV	19(12/7)	24(15/9)	51.70 ± 9.20	54.70 ± 5.30	3, 6	③
[23]	Vega et al., 2015	Spain	BM-MSCs	40 × 10^6^	II,III,IV	15 (9/6)	15 (10/6)	56.60 ± 9.24	57.33 ± 9.09	3, 6, 12	①②④⑦
[24]	Guptaet al.,l (1) 2016	India	BM-MSCs	25 × 10^6^	II,III,IV	10 (3/7)	10 (0/10)	58.10 ± 8.23	55.80 ± 6.78	1, 3, 6, 12	①②④⑦
	Guptaet al.,l (2) 2016	India	BM-MSCs	50 × 10^6^	II,III,IV	10 (2/8)	10 (0/10)	57.30 ± 9.45	55.80 ± 6.78	1, 3, 6, 12	①②④⑦
	Gupta et al., (3) 2016	India	BM-MSCs	75 × 10^6^	II,III,IV	10 (8/2)	10 (7/3)	55.00 ± 6.72	56.70 ± 5.19	1, 3, 6, 12	①②④⑦
	Gupta et al., (4) 2016	India	BM-MSCs	150 × 10^6^	II,III,IV	10 (5/5)	10 (7/3)	54.00 ± 6.73	56.70 ± 5.19	1, 3, 6, 12	①②④⑦
[25]	Matas et al., (1) 2019	Chile	UC-MSCs	20 × 10^6^	I,II,III	10 (4/6)	9 (4/5)	56.10 ± 6.80	54.80 ± 4.50	1, 2, 3, 4, 6, 9, 12	①②④⑦
	Matas et al., (1) 2019	Chile	UC-MSCs	20 × 10^6^	I,II,III	10 (5/5)	9 (4/5)	56.70 ± 4.10	54.80 ± 4.50	1, 2, 3, 4, 6, 9, 12	①②④⑦
[26]	Ha et al., 2018	China	UC-MSCs	5 × 10^6^	I,II,III	43 (12/32)	43 (11/32)	57.00 ± 3.20	56.20 ± 6.70	1, 3, 6, 12	②④⑤⑦
[27]	Yang et al., (1) 2017	China	UC-MSCs	30 × 10^6^	III,IV	15 (3/12)	16 (5/11)	70.60 ± 20.10	72.20 ± 17. 80	3, 6, 12	①
	Yang et al., (1) 2017	China	UC-MSCs	60 × 10^6^	III,IV	13 (3/10)	16 (5/11)	71.50 ± 16.30	72.20 ± 17. 80	3, 6, 12	②

① WOMAC, ② VAS, ③ KOOS, ④ MRI, ⑤ AKS, ⑥ SF-36, ⑦ AEs, ⑧ Lysholm; WOMAC, Western Ontario and McMaster Universities Osteoarthritis Index; VAS, Visual Analog Scale; KOOS, Knee Osteoarthritis Outcome Score; SF-36, Short Form-36 health survey; AKS, American Knee Society; AEs, adverse effects; AD-MSCs, adipose-derived mesenchymal stem cells, BM-MSCs, bone marrow mesenchymal stem cells; UC-MSCs, umbilical cord mesenchymal stem cells

### Risk of bias assessment

**Fig. 2:**
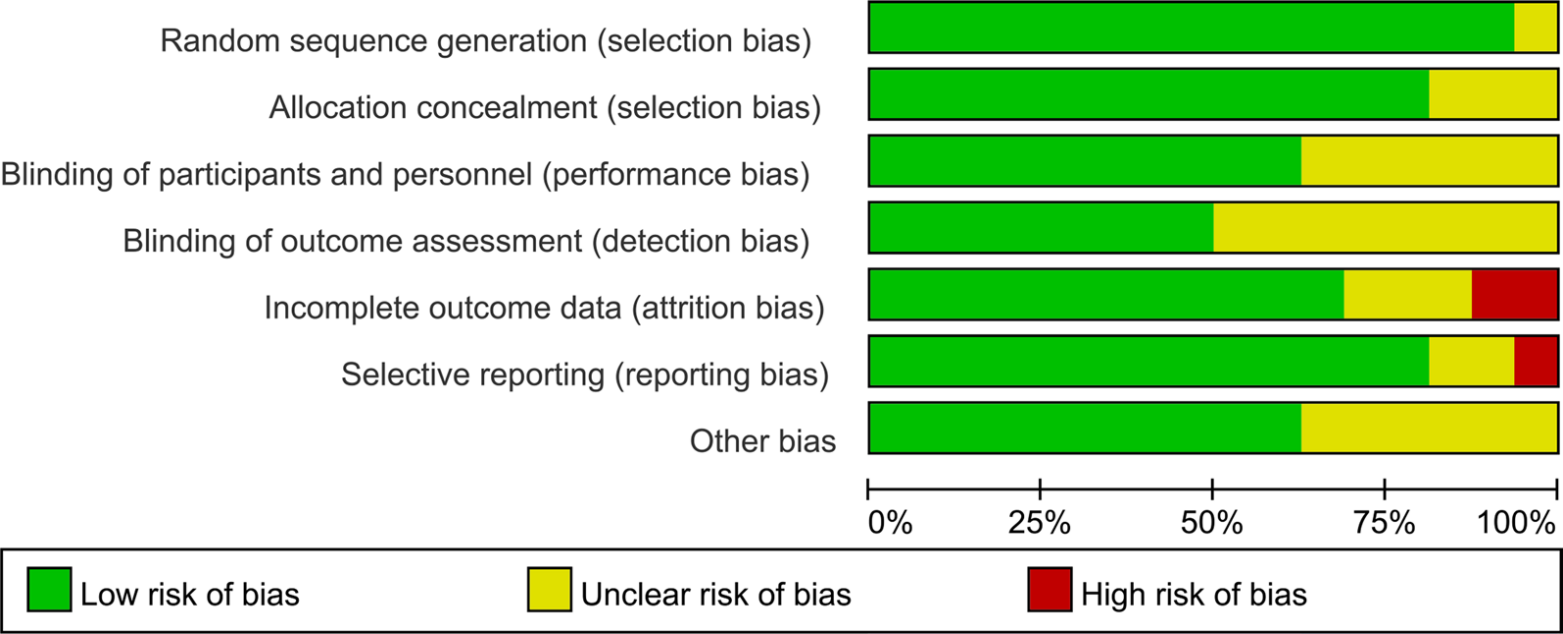
Risk of bias assessment diagram

The Cochrane Risk of Bias tool evaluated sixteen randomized controlled trials. The risk of bias is shown in Fig. 2.

### Primary outcomes

#### VAS score

##### VAS score at 3 months

**Fig. 3:**
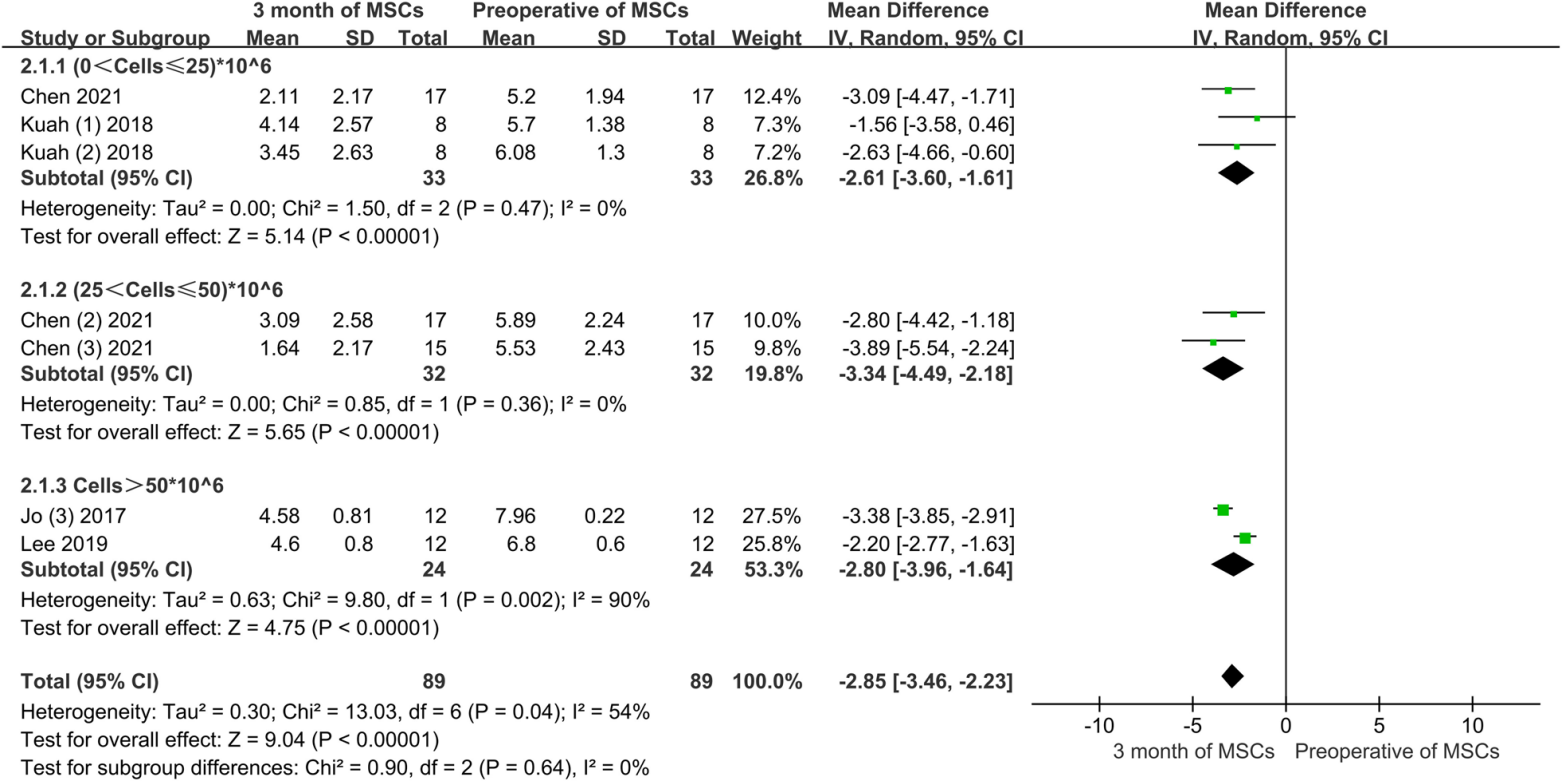
VAS scores from varying doses of AD-MSCs at 3 months

Conventional meta-analysis: two articles, which included three experimental groups with a total of 33 patients, provided information on VAS improvement at a low dose; one literature with two experimental arms, including 32 patients, provided moderate dose information on VAS improvement; two articles, including 24 patients, provided information on VAS improvement at high-dose. Conventional meta-analysis showed that regardless of low, moderate, or high doses of AD-MSCs, VAS scores improved significantly after 3 months of treatment compared with those before it. The pooled results did not show any significant differences among the varying doses of AD-MSCs (MD = − 2.85, 95% CI − 3.46 to 2.23, *P* > 0.05). In addition, the random effects model used for the heterogeneity test *I*^2^ = 54%, indicated high heterogeneity (Fig. 3).

**Fig. 4:**
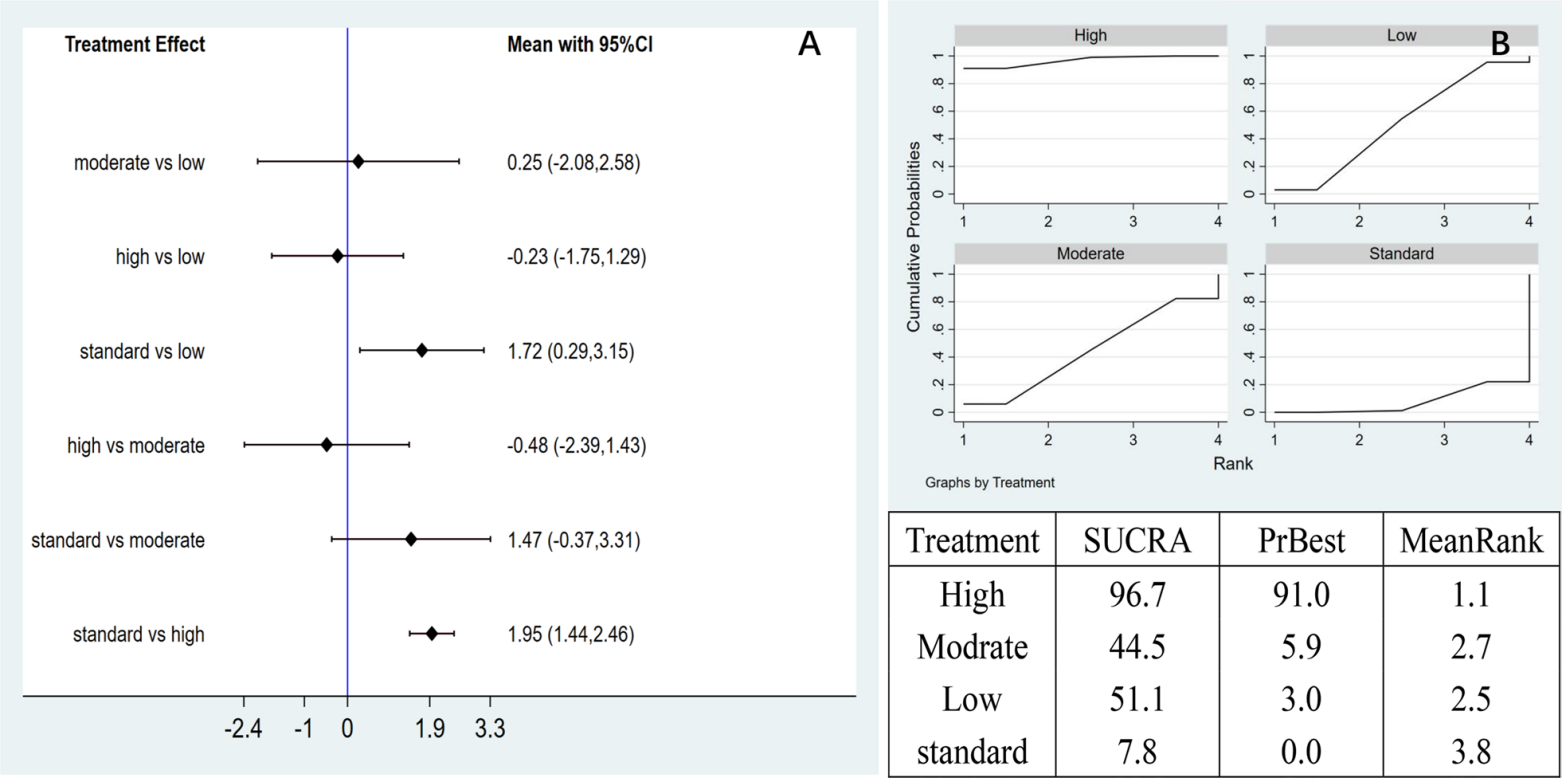
**A** Network meta-analysis of VAS scores at 3 months with varying doses of AD-MSCs; **B** SUCRA curve and area under the curve (%) of VAS score from varying doses of AD-MSCs for 3 months

Network meta-analysis: The network meta-analysis generated six pairwise comparison results, with statistically significant differences between the two groups. Compared with the control group, low-dose (MD = 1.72, 95% CI 0.29 to 3.15) and high-dose (MD = 1.95, 95% CI 1.44 to 2.46) AD-MSCs group showed significantly improved VAS pain score (Fig. 4A). The area under the SUCRA curve showed that the high-dose of AD-MSCs was the most effective (96.7%), followed by the low-dose (51.1%), moderate-dose (44.5%), and the control group (7.8%). This observation indicated that the high-dose AD-MSCs were potentially the best choice for improving VAS pain scores at 3 months (Fig. 4B).

##### VAS score at 6 months

**Fig. 5:**
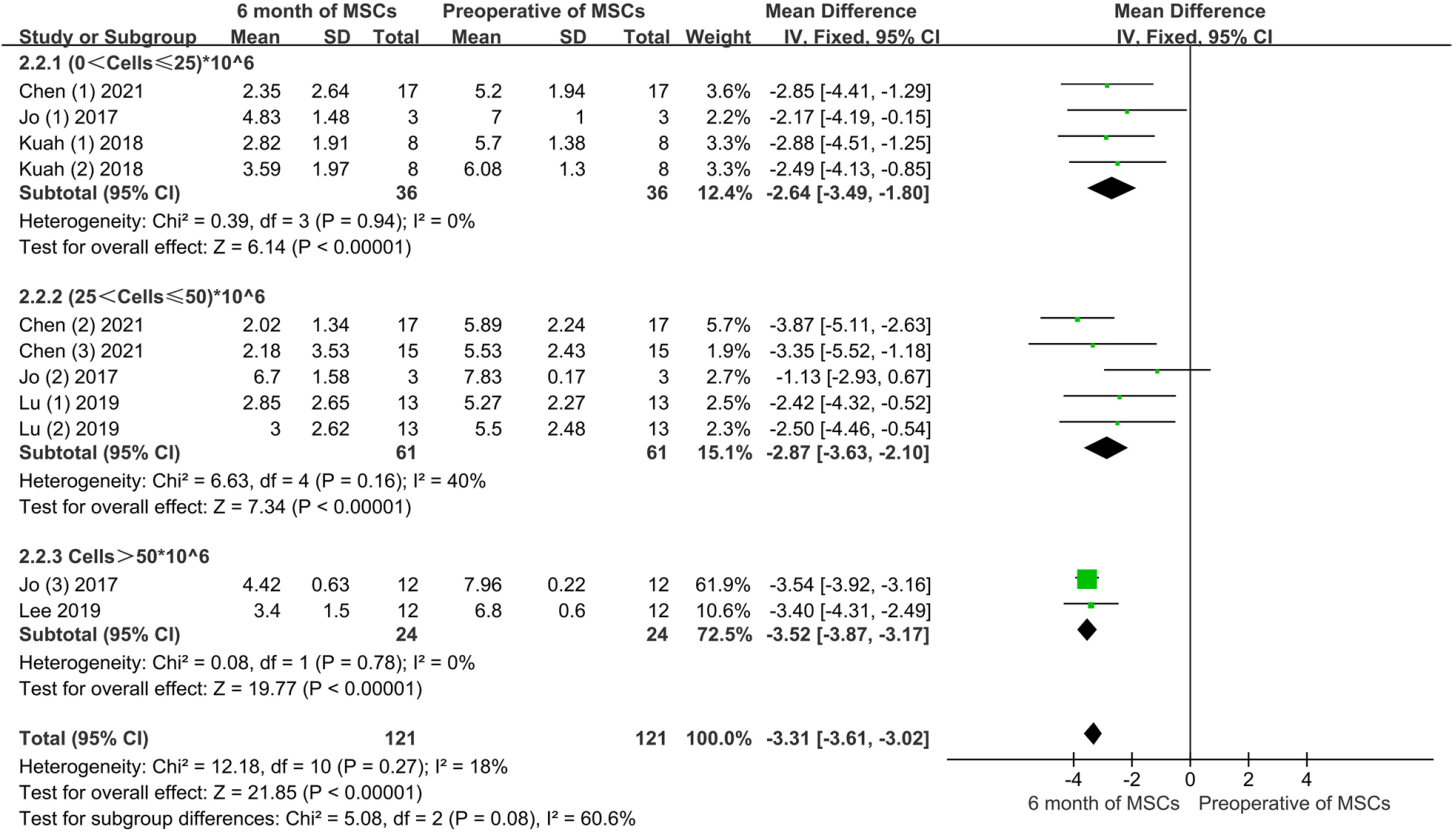
VAS scores of various doses of AD-MSCs at 6 months

Conventional meta-analysis: Three articles, including four experimental groups with a total of 36 patients, provided improvement information on low-dose of VAS; the three articles, including five experimental groups and 61 patients, provided input on moderate-dose of VAS improvement; two articles, including 24 patients, provided information on VAS improvement at high-dose. The conventional meta-analysis showed a significant improvement in the VAS score after 3 months of treatment, regardless of the low, moderate, or high dose of AD-MSCs. The pooled results showed no significant difference among varying doses of AD-MSCs (MD = − 3.31, 95% CI − 3.61 to − 3.02, *P* > 0.05). In addition, the heterogeneity test, with *I*^2^ = 18%, suggested low heterogeneity, so a fixed effects model was used (Fig. 5).

**Fig. 6:**
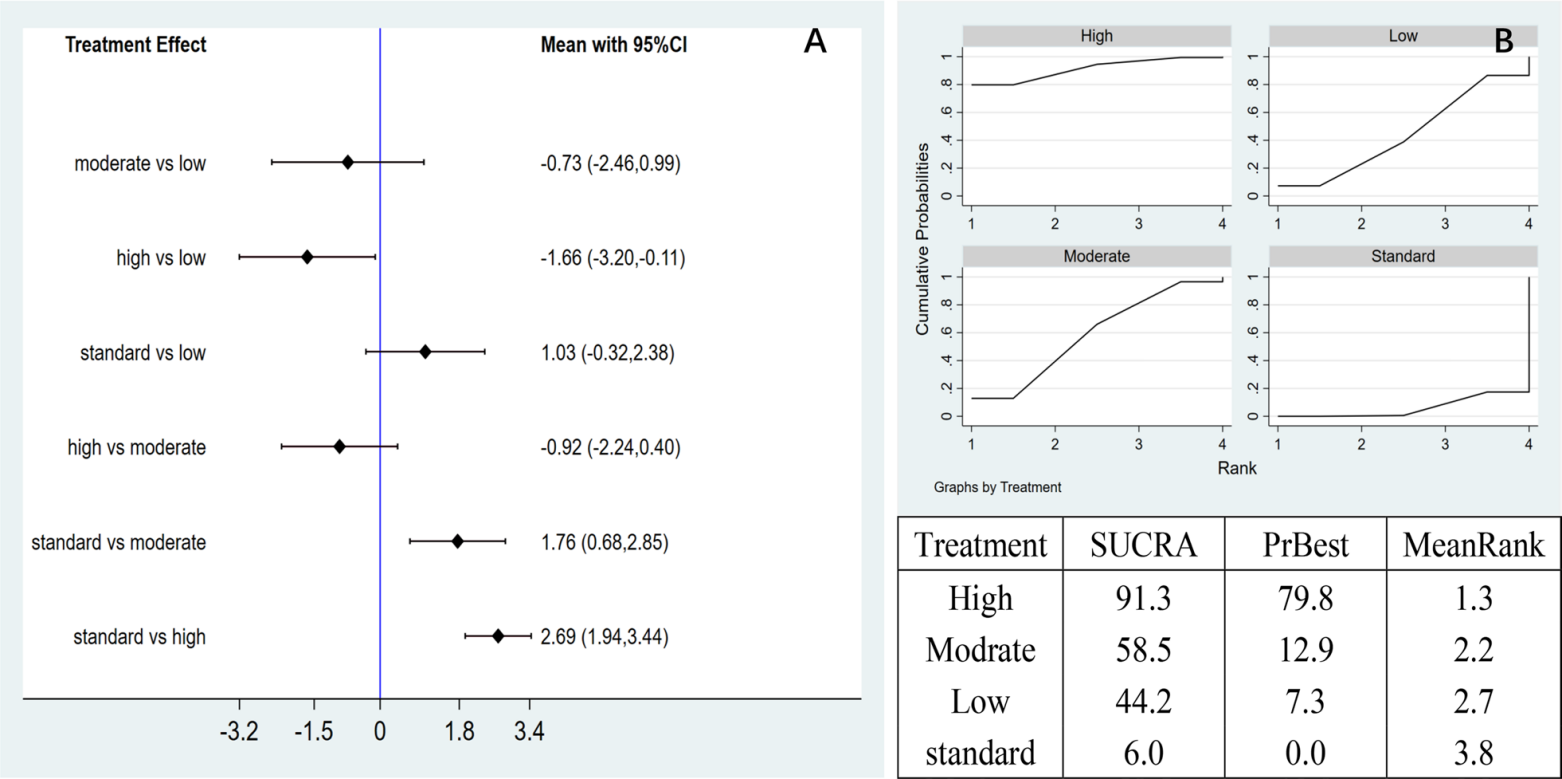
**A** Network meta-analysis of VAS scores at 6 months using varying doses of AD-MSCs; **B** SUCRA curve and area under the curve (%) of VAS score to varying doses of AD-MSCs for 6 months

Network meta-analysis: The network meta-analysis generated six pairwise comparison results, of which three groups had statistically significant differences. Compared with the control group, the high-dose group (MD = − 1.66, 95% CI − 0.30 to − 0.11) and the moderate-dose group (MD = 1.76, 95% CI 0.68 to 2.85) effectively reduced the VAS pain score. The high-dose group was better than the low-dose group (MD = − 1.66, 95% CI − 3.20 to − 0.11). (Fig. 6A). The area under the SUCRA curve revealed that the high-dose of AD-MSCs had the best efficacy (91.3%), followed by moderate-dose (58.5%), low-dose (44.2%), and control group (6.0%). This observation suggested that high-dose of AD-MSCs had more potential to improve VAS pain scores at 6 months (Fig. 6B).

##### VAS score at 12 months

**Fig. 7:**
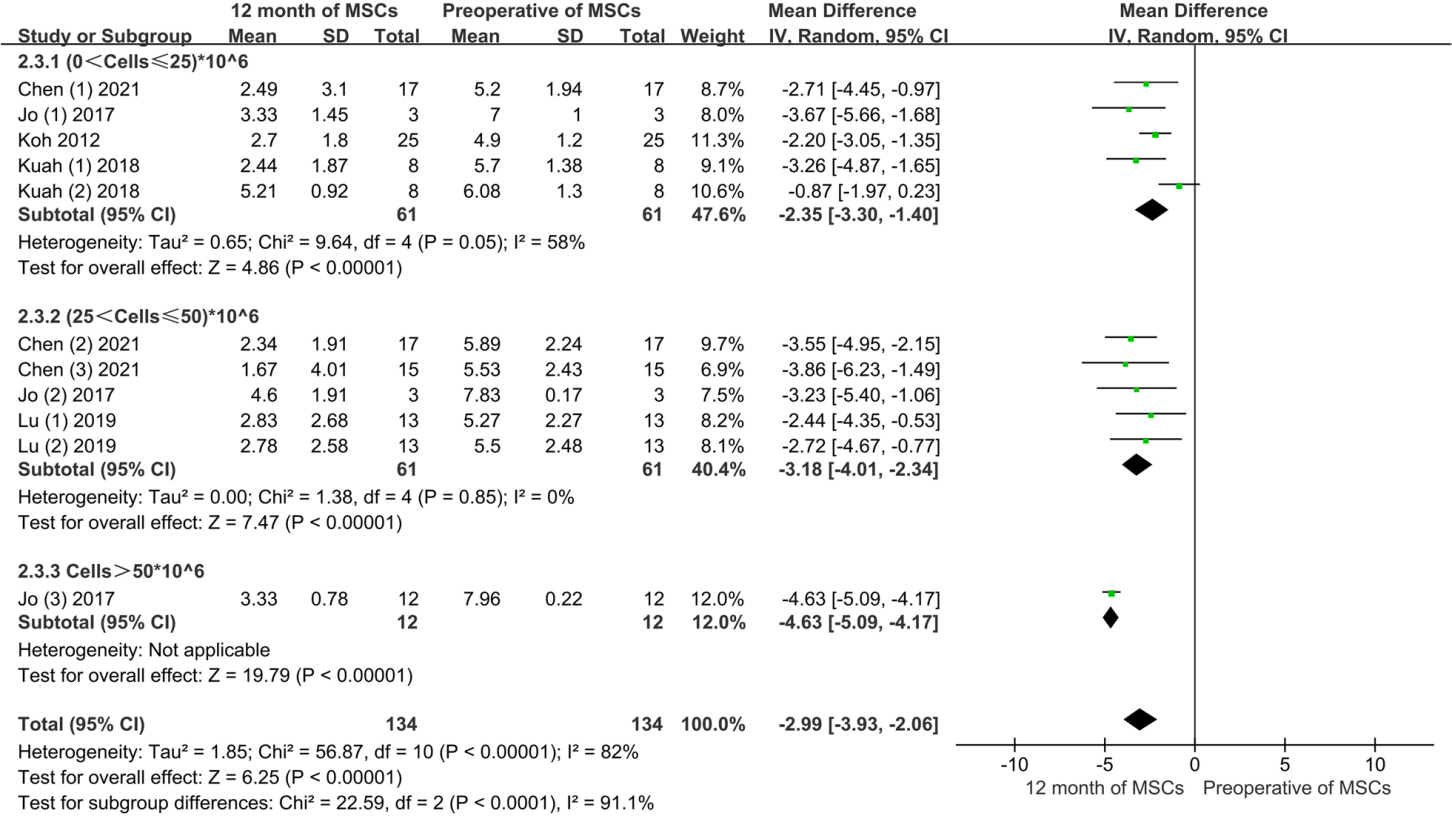
VAS scores of varying doses of AD-MSCs at 12 months

Conventional meta-analysis: Four articles, including five experimental groups and 61 patients, providing information on the improvement of VAS with low-dose; three articles, with five experimental groups and 61 patients, provided information on moderate-dose of VAS improvement; one article with 12 patients provided information on VAS improvement at high-dose. The conventional meta-analysis showed that the VAS score significantly improved after 3 months of treatment, regardless of the low, moderate, or high dose of AD-MSCs. The pooled results showed significant differences in the efficacies of the varying doses of AD-MSCs (MD = − 2.99, 95% CI − 3.93 to 2.06, *P* < 0.05). In addition, the heterogeneity test, *I*^2^ = 82%, suggested considerable heterogeneity, warranting using a random effects model (Fig. 7).

**Fig. 8:**
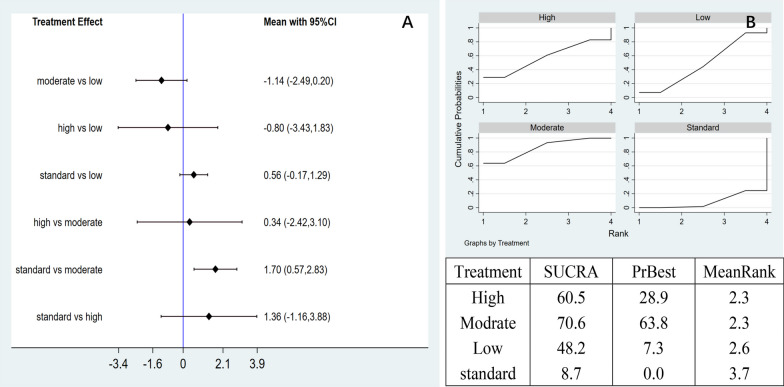
**A** Network meta-analysis of VAS scores at 12 months with varying doses of AD-MSCs; **B** SUCRA curve and area under the curve (%) of VAS score to varying doses of AD-MSCs for 12 months

Network meta-analysis: A total of six pairwise comparison results were generated in the network meta-analysis, and one of them was statistically significant. Compared with the control group (MD = 1.70, 95% CI 0.57 to 2.83), the moderate-dose group could effectively reduce the VAS pain score (Fig. 8A). According to the area under the SUCRA curve, the moderate-dose had the best efficacy (70.6%), followed by high-dose (60.5%), low-dose (48.2%), and control group (8.7%). This suggests that moderate-dose of AD-MSCs may be the best choice for improving VAS pain scores at 12 months (Fig. 8B).

#### WOMAC score

##### WOMAC score at 3 months

**Fig. 9:**
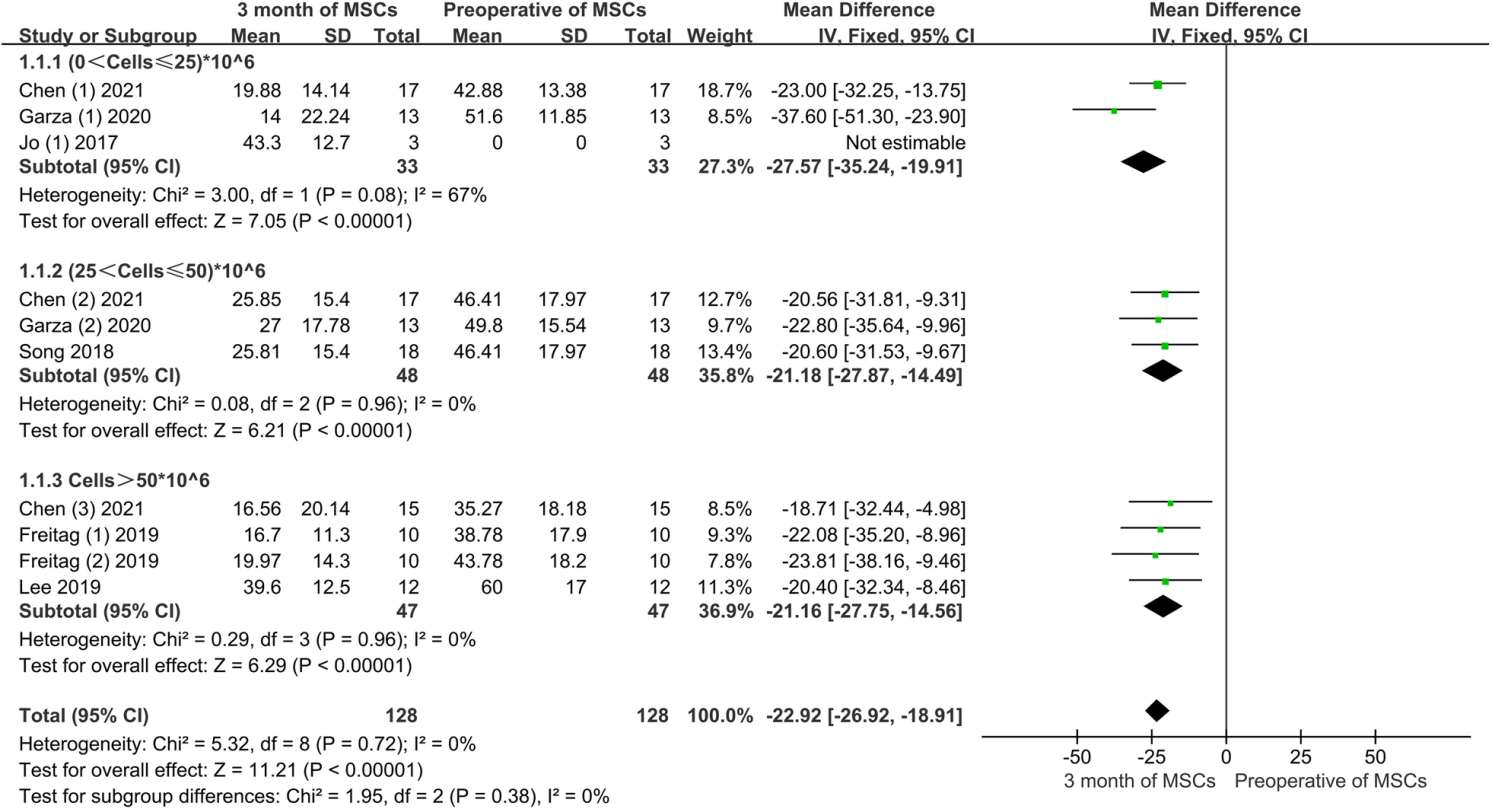
WOMAC scores of MSCs at 3 months for varying doses

Conventional meta-analysis: Three articles, including 30 patients, provided information on the improvement of WOMAC score at low doses; three studies, including 48 patients, provided information on the progress of WOMAC at moderate-dose. Three articles, including four experimental groups and 47 patients, provided information on WOMAC improvement at high-dose. Conventional meta-analysis showed that the WOMAC score significantly improved after 3 months of AD-MSC treatment, regardless of the low, medium, or high doses. The pooled results showed no significant difference between varying doses of AD-MSCs (MD = − 22.92, 95% CI − 26.91 to − 18.92, *P* > 0.05). In addition, the heterogeneity test, *I*^2^ = 0%, suggested no heterogeneity, leading us to use the fixed effects model (Fig. 9).

**Fig. 10:**
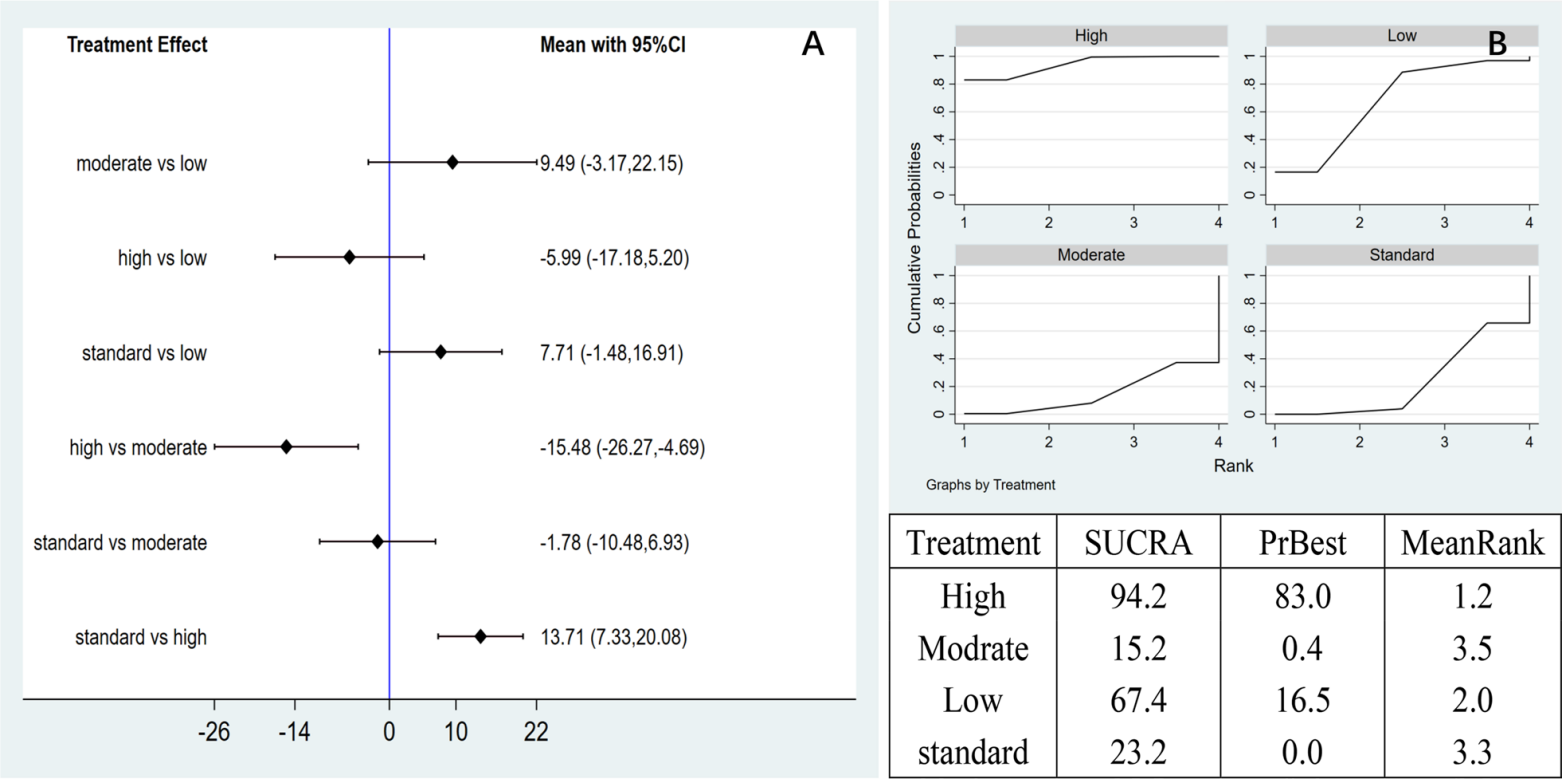
**A** Network meta-analysis of WOMAC scores at 3 months with varying doses of AD-MSCs; **B** SUCRA curve and area under the curve (%) of WOMAC score at 3 months with varying doses of AD-MSCs

Network meta-analysis: a total of six pairwise comparison results were generated in the network meta-analysis, with two groups showing significant differences. Compared with moderate-dose (MD = − 15.48, 95% CI − 26.27 to − 4.69) and control group (MD = 13.71, 95% CI 7.33 to 20.08), high-dose AD-MSCs significantly reduced WOMAC score (Fig. 10A). The SUCRA curve showed that the high dose of AD-MSCs was the best (94.2%), followed by the low-dose (67.4%), the control group (23.6%), and the moderate-dose (15.2%). This finding indicated the high-dose AD-MSCs might be the best choice for improving WOMAC scores at 3 months (Fig. 10B).

##### WOMAC score at 6 months

**Fig. 11:**
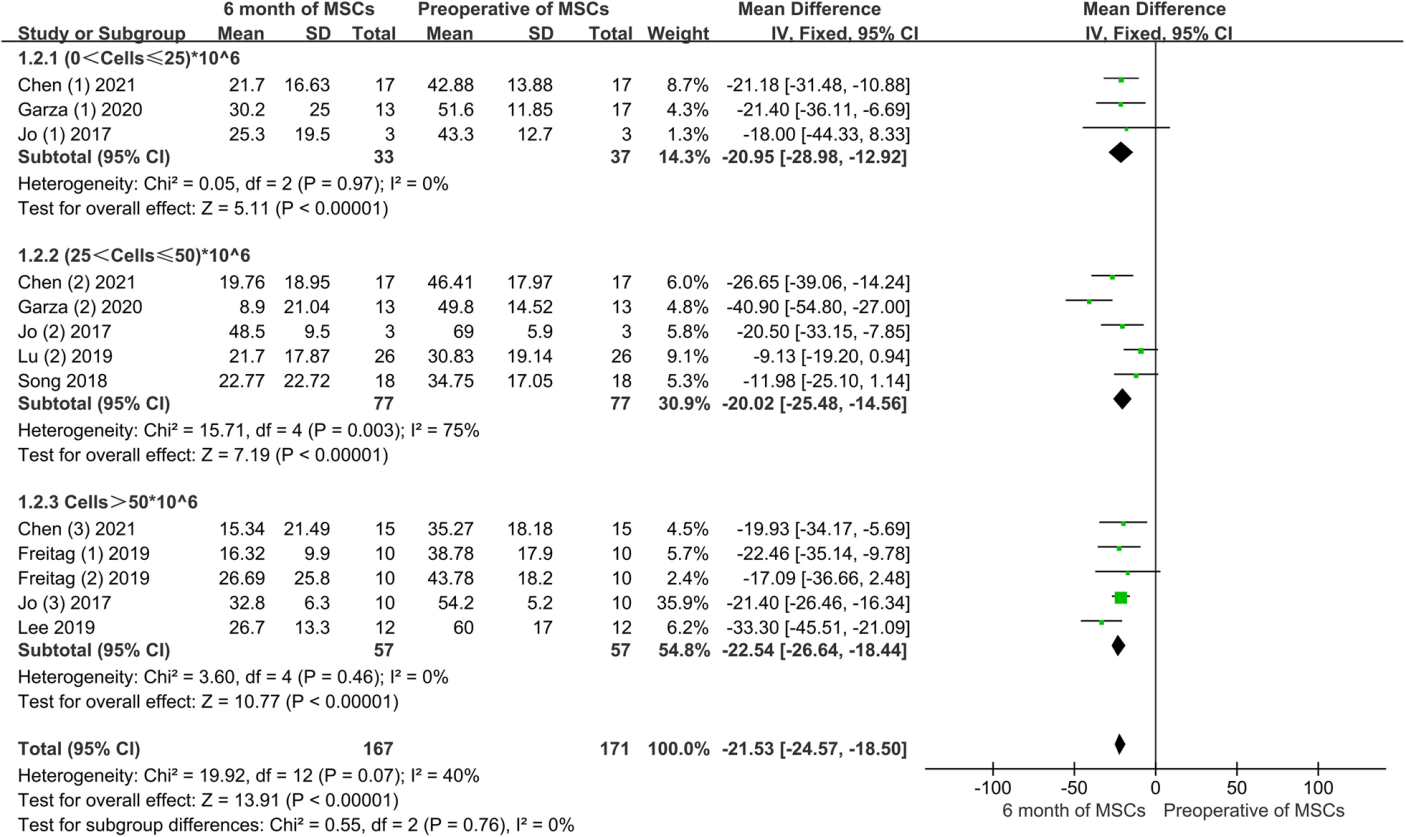
The WOMAC scores of MSCs at 6 months for varying doses

Conventional meta-analysis: three studies, including 33 patients, provided information on WOMAC improvement at a low dose; 5 studies, including 77 patients, provided information on WOMAC improvement at a moderate dose. Four articles, including five experimental groups and 57 patients, provided information on improving WOMAC at high doses. Conventional meta-analysis showed that the WOMAC score was significantly improved after 3 months of treatment, regardless of the low, moderate, or high dose of AD-MSCs. The pooled results showed that there was no significant difference between varying doses of AD-MSCs (MD = − 2.85, 95% CI − 3.46 to − 2.23, *P* > 0.05). In addition, the heterogeneity test, *I*^2^ = 54%, suggested high heterogeneity, leading us to use a random effects model (Fig. 11).

**Fig. 12:**
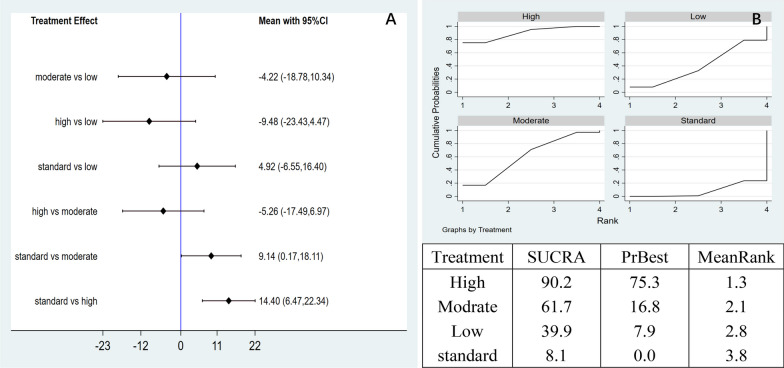
**A** Network meta-analysis of WOMAC scores at 6 months with varying doses of AD-MSCs; **B** SUCRA curve and area under the curve (%) of WOMAC score at 6 months with varying doses of AD-MSCs

Network meta-analysis: the network meta-analysis generated six pairwise comparison results, with significant differences in the two groups. Compared with the control group, the high-dose group (MD = 14.40, 95% CI 6.47 to 22.34) and the moderate-dose group (MD = 9.14, 95% CI 0.17 to 18.11) had a statistically significant difference (Fig. 12A). According to the area under the SUCRA curve, the efficacy of high-dose AD-MSCs was the best (90.2%), followed by the moderate-group (61.7%), low-dose (39.9%), and control dose (8.1%). This observation indicated that high-dose AD-MSCs might be the best choice to improve WOMAC score at 6 months (Fig. 12B).

##### WOMAC score at 12 months

**Fig. 13:**
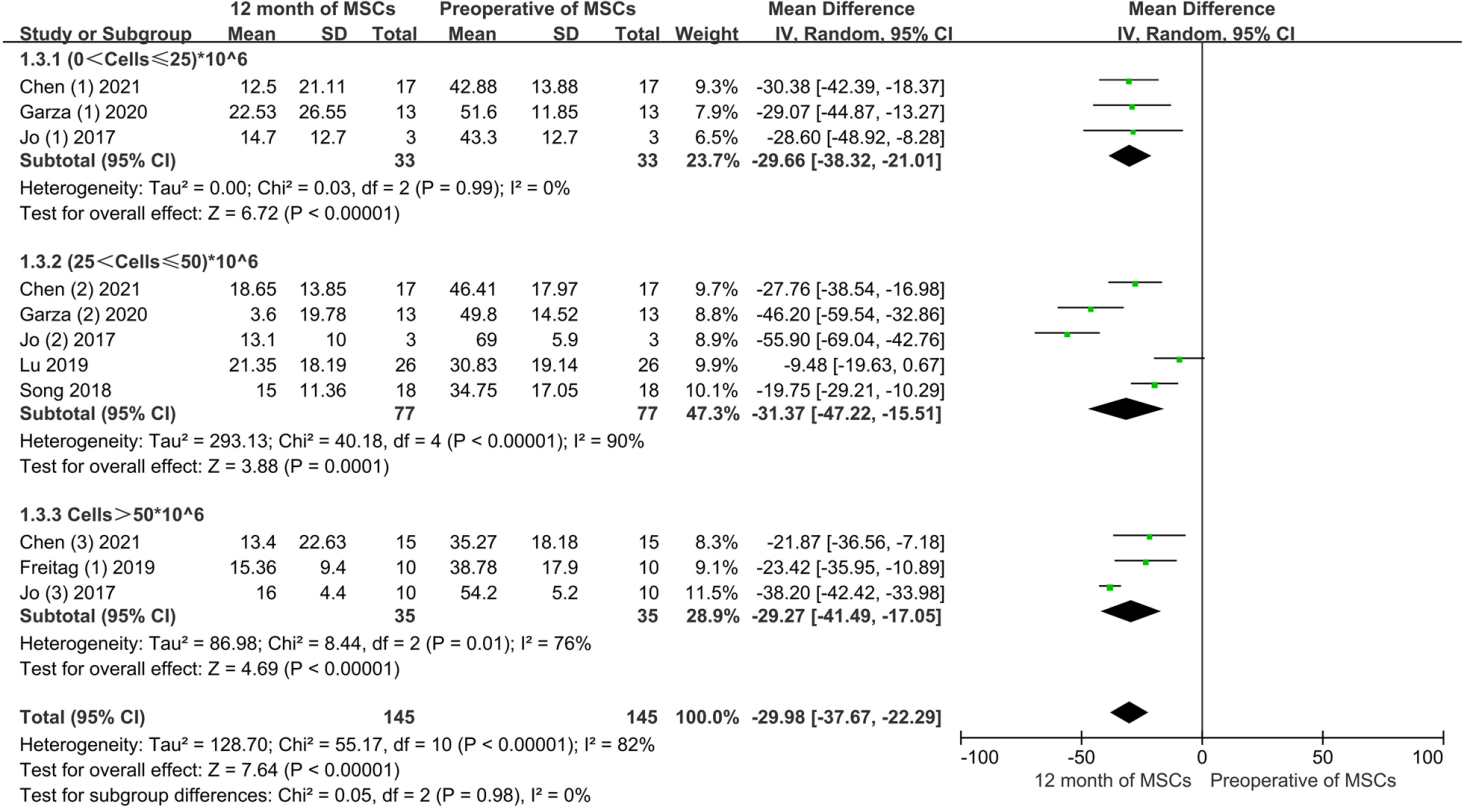
WOMAC scores of MSCs at 12 months for varying doses

Conventional meta-analysis: three studies, including 33 patients, provided information on WOMAC improvement at low doses; five studies, including 77 patients, provided information on WOMAC improvement at moderate doses; three articles, including 35 patients, provided information on WOMAC improvement at high doses. The conventional meta-analysis showed that the VAS score was significantly improved after 3 months of treatment, regardless of the low, moderate, or high doses of AD-MSCs. The pooled results showed no significant difference between varying doses of AD-MSCs (MD = − 29.98, 95% CI − 37.67 to − 22.29), *P* > 0.05). In addition, the heterogeneity test, *I*^2^ = 82%, suggested high heterogeneity, leading us to use a random effects model (Fig. 13).

**Fig. 14:**
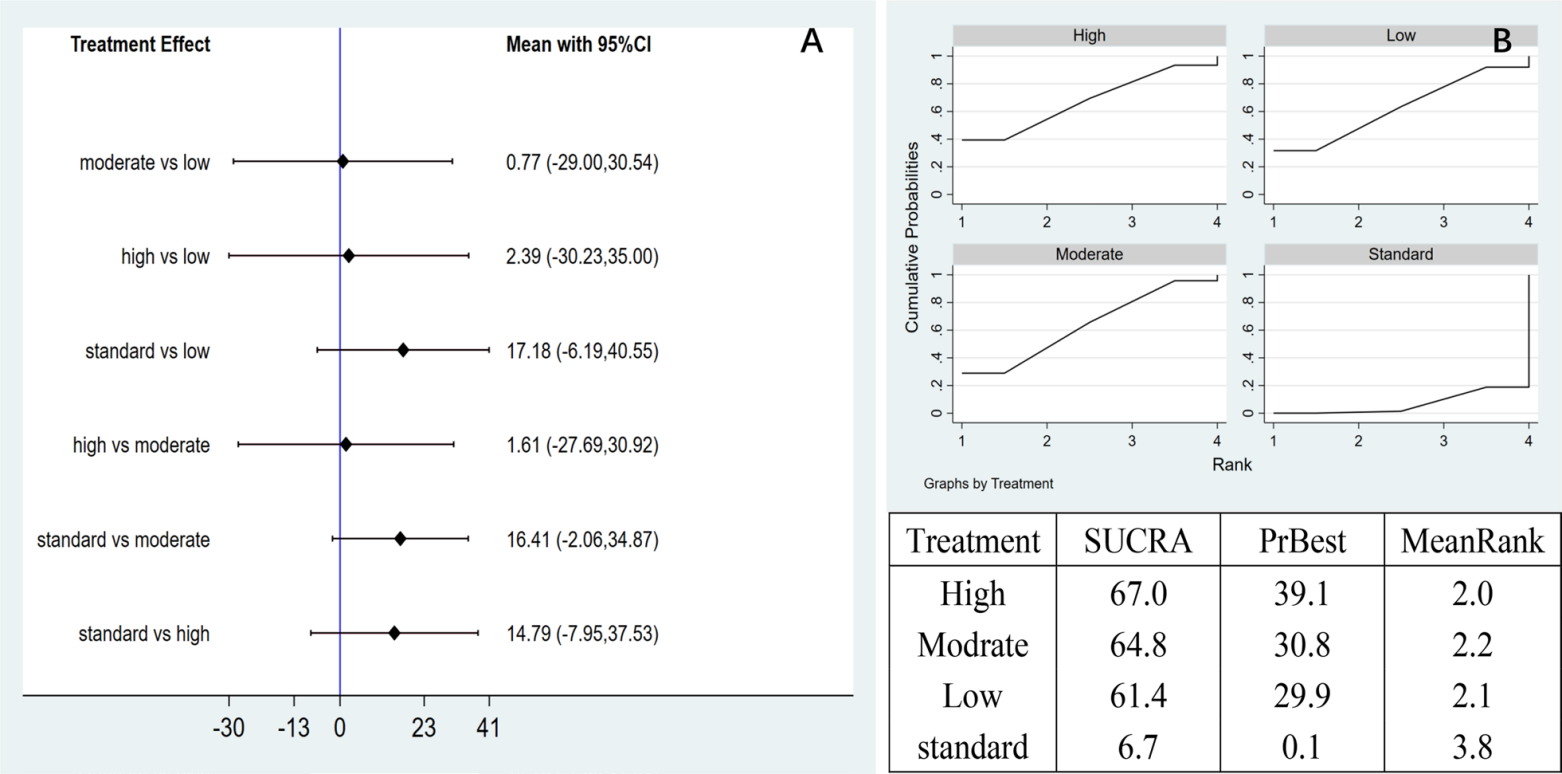
**A** Network meta-analysis of WOMAC scores at 12 months with varying doses of Ad-MSCs; **B** SUCRA curve and area under the curve (%) of WOMAC score at 12 months with varying doses of AD-MSCs

Network meta-analysis: The network meta-analysis produced six pairwise comparison results with no significant differences (Fig. 14A). The SUCRA curve, high-dose AD-MSCs had the best efficacy (67.0%), followed by moderate-dose (64.8%), low-dose (61.4%), and control group (6.7%). This suggests that high-dose of AD-MSCs may be the best option for improving WOMAC scores at 12 months (Fig. 14B).

#### SUCRA column analysis chart

**Fig. 15:**
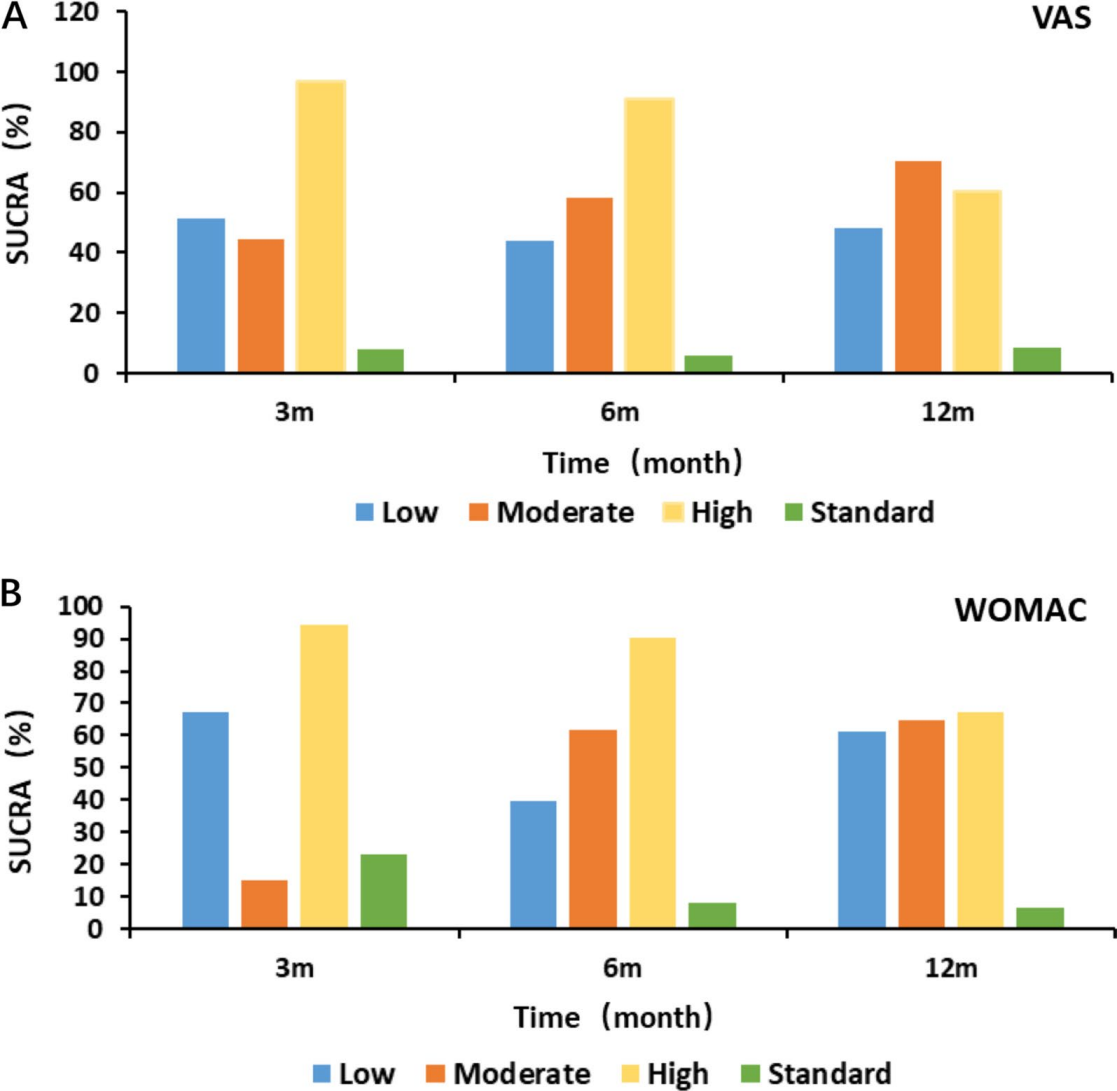
**A** VAS scores of AD-MSCs at the same dose at different periods; **B** WOMAC scores of the same dose of AD-MSCs at different periods

We plotted the area under the SUCRA curve for VAS and WOMAC from the network meta-analysis through a column plot (Fig. 15). Compared with the control group, all three cell doses can significantly reduce WOMAC and VAS scores, and the efficacy of intra-articular injection of AD-MSCs is worthy of recognition. From Fig. 15A: The improvement in WOMAC at 3, 6, and 12 months was better in the high-dose group than in the moderate-dose and low-dose groups. From Fig. 15B: The high-dose group had a dominant effect on VAS improvement at 3 and 6 months. At 12 months, the improvement of VAS in the moderate-dose group was better than that in the high-dose group and the low-dose group, but there was no significant difference in the improvement of VAS among the three groups.

#### Adverse events

**Fig. 16:**
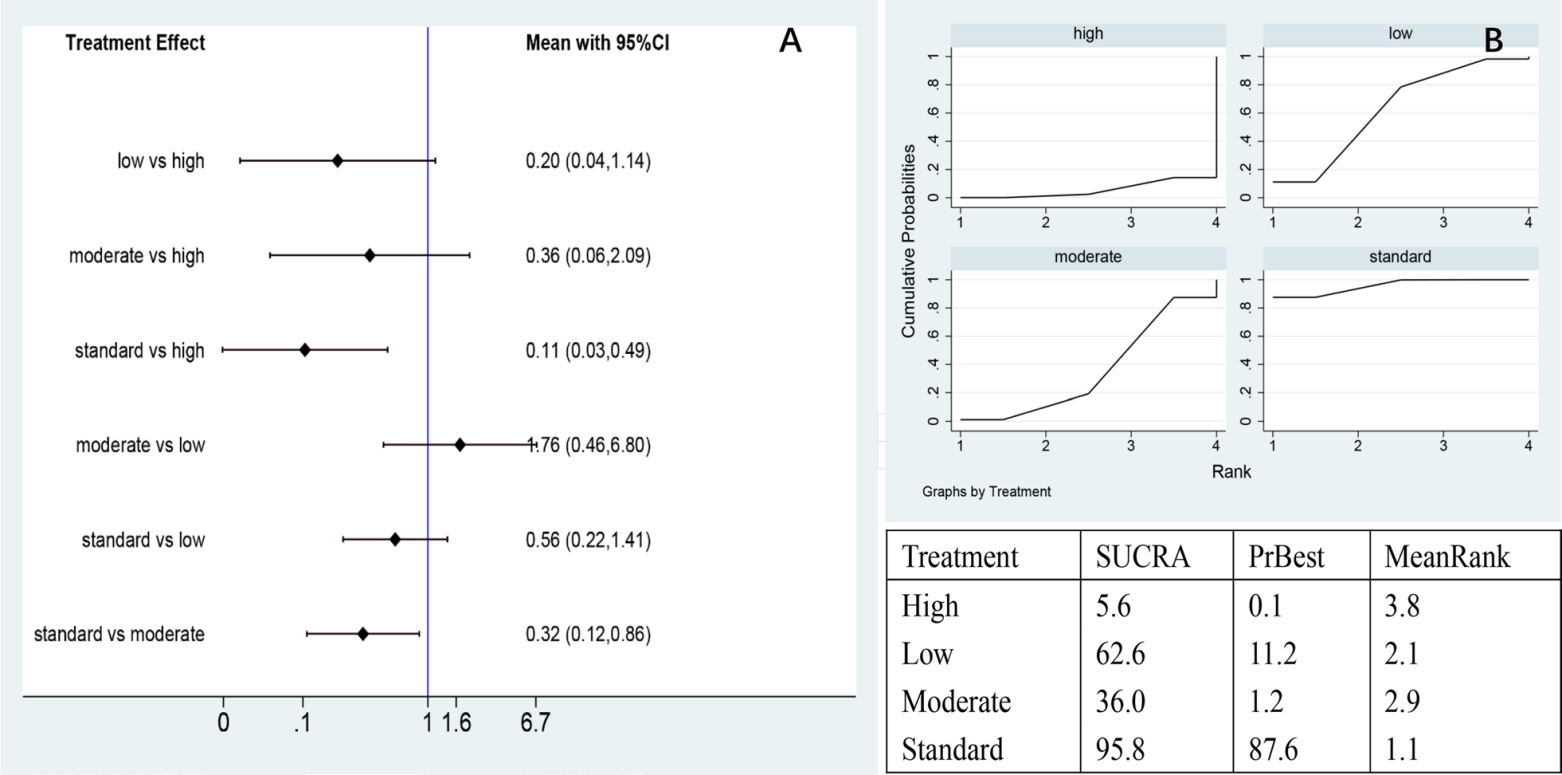
**A** Network meta-analysis of adverse events for varying doses of AD-MSCs; **B** SUCRA curve and area under the curve (%) for adverse events of varying doses of AD-MSCs

The network meta-analysis yielded a total of six pairwise comparisons, two of which had significantly fewer adverse events in the control group compared with the high-dose (MD = 0.11, 95% CI = [0.03; 0.49]), medium doses (MD = 0.32, 95% CI 0.12 to 0.86) (Fig. 16A). The ranking was performed based on the area under the SUCRA curve, indicating the control group to be the optimal one (95.8%), and the others were, in order, low-dose (62.6%), medium doses (36.0), and high-dose (5.6%). The results suggested that the incidence of adverse events increased with the number of cells (Fig. 16B).

#### Subgroup analysis VAS scores of different MSCs sources at the same doses

**Fig. 17:**
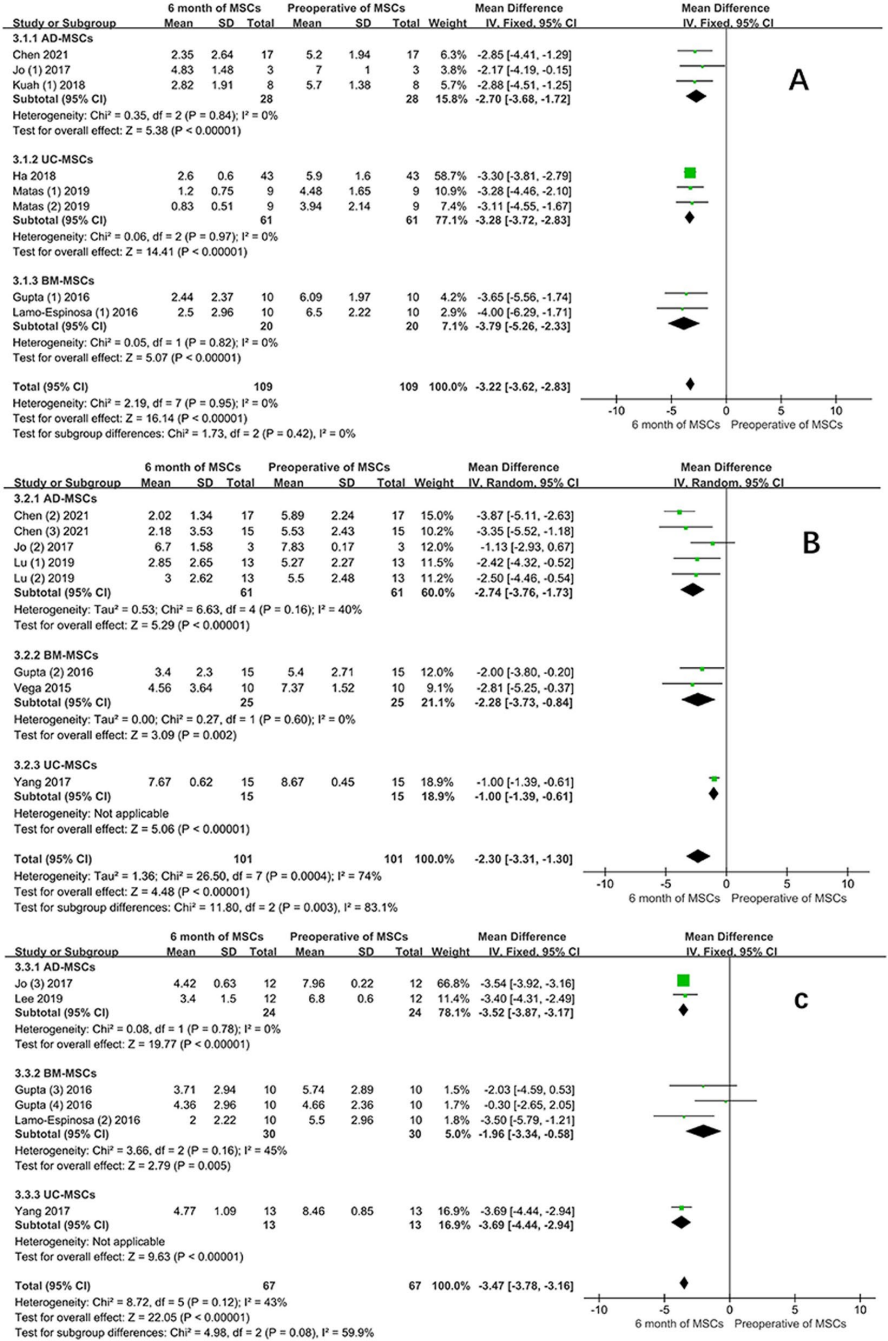
VAS scores of different MSCs derived from the same dose; **A** Low-dose group; **B** moderate-dose group; **C** high-dose group

Subgroup analysis of MSCs derived from different tissues at the same dose was performed to evaluate the changes in VAS scores of AD-MSCs, UC-MSCs, and BM-MSCs at 6 months. Figure 17 depicts that MSCs of different origins all had noticeable improvements in VAS scores, with no significant difference between the three various tissue sources of MSCs in the low and high-dose groups (*P* > 0.05). However, significant difference was observed in the moderate dose (*P* < 0.05).

#### Evidence network diagram

Most of the literature included in this study directly compared single-cell doses of AD-MSCs and the control group. This kind of analysis left a lack of evidence for direct comparison between varying doses of AD-MSCs, leaving scope for only indirect comparisons. Therefore, most of the evidence network does not have closed loops. The complete evidence network diagram is provided in Appendix 2.

#### Analysis of publication bias

The funnel plots for each outcome indicator showed that all the plots were symmetrical, and the study results were not affected by the publication bias of the included literature. The complete funnel plots are provided in Appendix 3.

## Discussion

A total of 16 RCTs were included in this study, of which 8 RCTs pertain to AD-MSCs, encompassing 15 experimental groups and 192 KOA patients. The conventional meta-analysis was used to analyze the effect of different doses of AD-MSCs on KOA, and the efficacy of different doses of AD-MSCs was ranked by network meta-analysis. It was found that AD-MSCs significantly improve the pain and knee function scores of KOA patients in the 3,6, and 12-month follow-ups compared with control group, and the therapeutic effects of different doses are different, with the high-dose group showing the best therapeutic effect. Therefore, in practical treatment, it is more recommended to use high doses of AD-MSCs for better therapeutic effects. The results of this network meta-analysis are consistent with those of other studies, showing that the treatment effect of the high-dose group is better than that of the moderate and low-dose groups. For example, Jo et al. [17] found that the efficacy of high-dose AD-MSCs (100 × 10^6) was better than that of low-dose (10 × 10^6) and moderate-dose (50 × 10^6), which could significantly improve the pain and joint function of patients. From Fig. 15, we can see that the improvement of VAS and WOMAC in the high-dose group was most significant at 3 and 6 months, while it was similar to that in the moderate-dose group and low-dose group at 12 months. We believe that VAS and WOMAC are subjective evaluation indicators, and high doses can relieve the symptoms in a short time, with high early satisfaction. However, with the time moving forward, the occurrence of knee pain symptoms in the high-dose group magnified the patients' sensitivity, so the sense of fall was significantly higher than that in the moderate and low-dose groups. Of note, our results showed that the incidence of adverse events was positively correlated with the cell dose, and the adverse reactions were mostly pain and swelling at the injection site in the first few days after intra-articular injection. No serious adverse reactions were reported. If the side effects are considered, low or moderate-dose may be considered.

Mesenchymal stem cells (MSCs) are pluripotent stem cells with the potential to self-renew and differentiate into different cell types, which have been widely used in the treatment of various diseases. KOA is a disease of the joints characterized by damage and inflammation of the joint cartilage. MSCs can be used to treat knee inflammation through a variety of mechanisms, including anti-inflammatory, cartilage tissue repair, reducing apoptosis and promoting angiogenesis [28, 29]. Specifically, MSCs can secrete many anti-inflammatory cytokines, such as IL-10, TGF-β and IL-1Ra, which can inhibit the inflammatory response and reduce the symptoms of knee arthritis. In addition, MSCs can differentiate into chondrocytes and secrete a large number of cartilage matrix components to help repair damaged cartilage tissue. MSCs can also promote joint tissue repair by inhibiting apoptosis and promoting angiogenesis. In conclusion, MSCs play a role in the treatment of KOA through multiple mechanisms, which can promote cartilage repair and regeneration, inhibit inflammatory response, regulate the immune system and promote angiogenesis, thereby reducing pain and improving joint function.

The extensive use of MSCs intra-articular injection in the treatment of KOA has corroborated its efficacy [30]. However, the clinical application of MSCs still contends with two primary practical issues: the tissue source of MSCs and the standardization of cell injection dose. In recent years, a growing body of research has elucidated the efficacy of MSCs from different tissue sources in treating KOA. For example, a network meta-analysis by Wei et al. [8] showed that AD-MSCs were the most effective in relieving pain among all MSCs. At the same time, UC-MSCs were the most effective in functional improvement [8]. Jeyaraman et al. [7] showed through meta-analysis that AD-MSCs are more effective inimproving the VAS and WOMAC scores of KOA patients than BM-MSCs. Basic research experiments have demonstrated AD-MSCs to have more vital proliferation ability and tolerance to hypoxia in the joint cavity than BM-MSCs [31]. Moreover, in vitro expansion experiments, have confirmed that AD-MSCs are more advanced in genetic stability than BM-MSCs [32]. Toward investigating the second problem, there is still insufficient clinical evidence to prove the standard therapeutic dose. To our knowledge, network meta-analyses on varying doses of MSCs in treating KOA are lacking. Only some conventional meta-analyses have mentioned the efficacy of varying doses of MSCs in subgroup analysis [9]. Therefore, this paper summarizes the cell doses, divided into three groups, required by previous studies in treating KOA with MSCs, by directly or indirectly comparing the therapeutic effects of different cell doses.

Treating KOA with adipose-derived MSCs began in 2014 [33]. Adipose tissue is increasingly used as a source of MSCs, benefited from its easy availability and ability to produce a large number of relatively uniform MSCs, making it an ideal choice for clinical applications. Therefore, the current treatment of KOA with MSCs is mainly from adipose tissue, and a large number of clinical evidences have accumulated to confirm the efficacy of AD-MSCs in the treatment of KOA, which is the reason why AD-MSCs were selected for network meta-analysis.

This paper carries out network meta-analysis, which overcomes the shortcomings of traditional meta-analysis that fails to compare multiple treatment groups simultaneously. However, it has certain limitations. This study only included all RCTs, single-center, and small sample clinical studies and lacked multi-center and large sample size trials. Standard cell injection doses are still lacking in clinical practice. In this study, we divided the cell doses into low, moderate, and high levels, which hinders us from obtaining a standardized and individualized treatment plan. Other factors, such as the age of patients and severity of disease, may also affect the therapeutic effect. Most of the studies included patients with Kellgren–Lawrence grade 2–3, and a small number of studies included patients with Kellgren–Lawrence grade 4 [14, 15]. Moreover, the majority of these studies focused on patients with a mean age ranging from 50 to 65, whereas a small number involved patients over the mean age of 65. It remains unclear whether OA patients with different Kellgren–Lawrence grade and age groups share different responsiveness to MSCs treatment. Notably, we included different strategies for identifying MSCs in the literature (Table 2). The majority of the studies used the classical surface marker for identification (CD73, CD90, CD105), while fewer studies used differentiation experiments for tangible validation. This lack of characterization may create potential heterogeneity in cell origin and provide additional bias to our meta-analysis. Future clinical studies may need to enhance the identification of MSCs to meet uniform standards [34]. However, differentiation assays in vitro and in vivo often require an additional 2–3 weeks to perform, which may be difficult to achieve in clinical practice. In addition, the standardization of the cell preparation and production process would also affect the therapeutic effect of MSCs in patients, which is where clinical studies differ from preclinical studies. Our analysis indicated that some studies adhered to Good Manufacturing Practice (GMP) compliance (Table 2). However, others tended to rely on third-party companies for preparation [19], which might necessitate additional transit for the tissues, while some studies had onsite lab to conduct the extraction [12]. These may affect the therapeutic effect of the cells and thus create dosage heterogeneity. We were unable to perform additional subgroup analyses based on cell preparation methods or characterization criteria, which may be an additional limitation of our study.

**Table 2:** Characterization and preparation methods of the MSCs

References	Study	Country	Origin	Characterization standards	Preparation methods (location, method)	Compliance with GMP standards
[11]	Freitag et al., (1) 2019	Australia	AD-MSCs	Surface marker (s CD90+, CD73+, CD 105+, CD14−, CD19−, CD34−, CD45−)	Onsite laboratory, Enzymatic digestion	
[12]	Garza et al., (1) 2020	USA	AD-MSCs	Surface marker (CD45−, CD31−, CD34+)	N.A, Enzymatic digestion	
[13]	Koh et al., 2012	South Korea	AD-MSCs	NO	Operating room, Enzymatic digestion	
[14]	Lee et al., 2019	South Korea	AD-MSCs	Surface marker (purity: CD31, CD34, CD45; identity: CD73, CD90)	Laboratory, Enzymatic digestion	
[15]	Lu et al., 2019	China	AD-MSCs	Surface marker (Cd105+, CD73+, CD90+, CD45−, CD34−, CD14−, CD11b−, CD79a−, CD19−, HLA II−)	Laboratory, Medium culture	Yes
[16]	Jo et al., 2017	Korea	AD-MSCs	Surface marker (CD31, CD34, CD45; identity: CD73, CD90)	N.A, N. A	Yes
[17]	Kuah et al., 2018	Australia	AD-MSCs	NO	Medicine Centre, N. A	Yes
[18]	Chen et al., 2021	China	AD-MSCs	Surface marker(unspecified), tri-lineage differentiation	N.A, Enzymatic digestion	
[19]	Bastos et al., 2019	Portugal	BM-MSCs	NO	N.A, N. A	
[20]	Lamo‑Espinos et al., 2016	Spain	BM-MSCs	Surface marker (CD90+, CD73+, CD44+, CD34−, CD45−)	N.A, Medium culture	Yes
[21]	Emadedin et al., 2018	Iran	BM-MSCs	NO	N.A, N.A	
[22]	Vega et al., 2015	Spain	BM-MSCs	Surface marker (CD90+, CD73+, CD105+, CD166+, CD34−, CD45−, CD14−, CD19−, HLA-DR-)	Operating room, enzymatic digestion	
[23]	Guptaet al., 2016	India	BM-MSCs	Surface marker (CD90+, CD73+, CD105+, CD166+, CD34−, CD45−, CD14−, CD19−, CD133+, HLA-DR-)	N.A, enzymatic digestion	Yes
[24]	Matas et al., 2019	Chile	UC-MSCs	Surface marker (CD73+, CD90+, CD 105+, CD14−, CD34−, CD45−)	Laboratory, Medium culture	Yes
[25]	Ha et al., 2018	China	UC-MSCs	Surface marker CD19−, CD34−, CD45−, CD11b−, HLA-ABC-, CD29+, CD44+, CD73+, CD90+, CD105+	Central Laboratory, Enzymatic digestion	
[26]	Yang et al., 2017	China	UC-MSCs	NO	Central Laboratory, Enzymatic digestion	Yes

## Conclusions

Sixteen literatures were included in this study, eight of which were related to AD-MSCs in the treatment of KOA. Overall, it was found that both cell doses reduced pain and improved knee function in KOA patients and were significantly better than those in the control group. It was noted that superior results were achieved by the high-dose group in comparison to the moderate and low-dose groups, and the patients' pain and dysfunction indicators improved more significantly in the early stage. The high-dose group typically used twice as many cells or more than the moderate-dose group. Therefore, an ample cell doses may yield a greater therapeutic effect on KOA. In conclusion, the results of this network meta-analysis indicate that AD-MSCs is a promising treatment for knee OA, but it needs to be carefully considered in clinical application, and the potential risks and side effects still need to be noted. Due to the limitations of this meta-analysis, future studies need to further explore the efficacy and safety of different doses, and carry out large sample, multi-center, randomized controlled trials to ensure the reliability and promotion value of the research results.

## Data Availability

Not applicable.

## Appendix 1

### PubMed

#1: Osteoarthritis [Mesh Terms]

#2: (Osteoarthritis[Title/Abstract]) OR (Osteoarthritides[Title/Abstract]) OR (Osteoarthrosis[Title/Abstract]) OR (Osteoarthroses[Title/Abstract]) OR (Osteoarthritic[Title/Abstract]) OR (“Degenerative Arthritides”[Title/Abstract]) OR (“Degenerative Arthritis”[Title/Abstract]) OR (“Osteoarthrosis Deformans”[Title/Abstract]) OR (Arthrosis [Title/Abstract]) OR (Arthroses [Title/Abstract]) OR (OA[Title/Abstract])

#3: #1 OR #2

#4: (knee [Mesh Terms]) OR (knee [Title/Abstract]) OR (knees [Title/Abstract])

#5: “Mesenchymal Stem Cells” [Mesh Terms]

#6: (“stem cell” [Title/Abstract]) OR (“stem cells” [Title/Abstract]) OR (“stromal cell” [Title/Abstract]) OR (“stromal cells” [Title/Abstract]) OR (“progenitor cell” [Title/Abstract]) OR (“progenitor cells” [Title/Abstract]) OR (“bone marrow stromal cell” [Title/Abstract]) OR (“bone marrow stromal cells” [Title/Abstract]) OR (“bone marrow stromal stem cell” [Title/Abstract]) OR (“bone marrow stromal stem cells” [Title/Abstract]) OR (“Wharton Jelly Cells” [Title/Abstract]) OR (“Wharton’s Jelly Cells” [Title/Abstract]) OR (“adipose-derived stem cells” [Title/Abstract]) OR (“Adipose Derived Mesenchymal Stromal Cells”[Title/Abstract]) OR (“Adipose Tissue-Derived Mesenchymal Stromal Cells”[Title/Abstract]) OR (“Adipose Tissue Derived Mesenchymal Stromal Cells”[Title/Abstract]) OR (“Stromal Cells”[Title/Abstract]) OR (“Stromal Cell”[Title/Abstract]) OR (“Stem Cells”[Title/Abstract]) OR (“Stem Cell”[Title/Abstract]) OR (“MSC”[Title/Abstract])

#7: #5 OR #6 OR

#8: “Clinical Trials as Topic” [Mesh Terms]

#9: (“randomized controlled trial”[Publication Type]) OR (“controlled clinical trial”[Publication Type]) OR (randomized[Title/Abstract]) OR (placebo[Title/Abstract]) OR (randomly[Title/Abstract]) OR (trial[Title]) OR (double blind method) OR (double-blind) OR (single blind method) OR (triple blind method)

#10: #8 OR #9

#11: #3 AND #4 AND #7 AND #10

### Cochrane Library

#1: MeSH descriptor: [Osteoarthritis] explode all trees

#2: osteoarthritis:ab,ti OR osteoarthritides:ab,ti OR osteoarthrosis:ab,ti OR osteoarthroses:ab,ti OR osteoarthritic:ab,ti OR ‘degenerative arthritides’:ab,ti OR ‘degenerative arthritis’:ab,ti OR ‘osteoarthrosis deformans’:ab,ti OR arthrosis:ab,ti OR arthroses:ab,ti OR OA:ab,ti

#3: #1 OR #2

#4: knee:ab,ti OR knees:ab,ti

#5: #3 OR #4

#6: MeSH descriptor: [Mesenchymal Stem Cells] explode all trees

#7: (Mesenchymal Stem Cell):ab,ti OR (Mesenchymal Stem Cells):ab,ti OR (Mesenchymal Stromal Cell):ab,ti OR (Mesenchymal Stromal Cells):ab,ti OR (Mesenchymal Progenitor Cell):ab,ti OR (Mesenchymal Progenitor Cells):ab,ti OR (Bone Marrow Stromal Cell):ab,ti OR (Bone Marrow Stromal Cells):ab,ti OR (Bone Marrow Stromal Stem Cell’:ab,ti OR ‘Bone Marrow Stromal Stem Cells’:ab,ti OR ‘Wharton Jelly Cells’:ab,ti OR ‘Bone Marrow Mesenchymal Stem Cells’:ab,ti OR ‘Bone Marrow Stromal Cells):ab,ti OR (Bone Marrow Stromal Cell):ab,ti OR (Adipose-Derived Mesenchymal Stem Cells):ab,ti OR (Adipose Derived Mesenchymal Stem Cells):ab,ti OR (Adipose Tissue-Derived Mesenchymal Stem Cells):ab,ti OR (Adipose Tissue Derived Mesenchymal Stem Cells):ab,ti OR (Adipose-Derived Mesenchymal Stromal Cells):ab,ti OR (Adipose Derived Mesenchymal Stromal Cells):ab,ti OR (Adipose Tissue-Derived Mesenchymal Stromal Cells):ab,ti OR (Adipose Tissue Derived Mesenchymal Stromal Cells):ab,ti OR (Stromal Cells):ab,ti OR (Stromal Cell):ab,ti OR (Stem Cells):ab,ti OR (Stem Cell):ab,ti OR (MSC):ab,ti

#8: #6 OR #7

#9: #3 AND #5 AND #8

### Embase

#1: osteoarthritis/exp

#2: 'osteoarthritis':ab,ti OR 'osteoarthritides':ab,ti OR 'osteoarthrosis':ab,ti OR 'osteoarthroses':ab,ti OR 'osteoarthritic':ab,ti OR 'degenerative arthritides':ab,ti OR 'degenerative arthritis':ab,ti OR 'osteoarthrosis deformans':ab,ti OR 'arthrosis':ab,ti OR 'arthroses':ab,ti OR 'oa':ab,ti

#3: #1 OR #2

#4: knee:ab,ti OR knees:ab,ti

#5: ‘mesenchymal stem cell’/exp

#6: ‘Mesenchymal Stem Cell’:ab,ti OR ‘Mesenchymal Stem Cells’:ab,ti OR ‘Mesenchymal Stromal Cell’:ab,ti OR ‘Mesenchymal Stromal Cells’:ab,ti OR ‘Mesenchymal Progenitor Cell’:ab,ti OR ‘Mesenchymal Progenitor Cells’:ab,ti OR ‘Bone Marrow Stromal Cell’:ab,ti OR ‘Bone Marrow Stromal Cells’:ab,ti OR ‘Bone Marrow Stromal Stem Cell’:ab,ti OR ‘Bone Marrow Stromal Stem Cells’:ab,ti OR ‘Wharton Jelly Cells’:ab,ti OR ‘adipose-derived stem cells’:ab,ti OR ‘Adipose Derived Mesenchymal Stromal Cells’:ab,ti OR ‘Adipose Tissue-Derived Mesenchymal Stromal Cells’:ab,ti OR ‘Adipose Tissue Derived Mesenchymal Stromal Cells’:ab,ti OR ‘Stromal Cells’:ab,ti OR ‘Stromal Cell’:ab,ti OR ‘Stem Cells’:ab,ti OR ‘Stem Cell’:ab,ti OR ‘MSC’:ab,ti

#7: #5 OR #6

#8: ‘Clinical Trial (topic)’/exp

#9: ‘randomized controlled trial’:pt OR ‘controlled clinical trial’:pt OR randomized:ab OR placebo:ab OR randomly:ab OR trial:ti

#10: #8 OR #9

#11: #3 AND #4 AND #7 AND #10

### Web of science

#1: TS = (Osteoarthritis OR osteoarthrities OR Osteoarthrosis OR osteoarthrosis OR Osteoarthritic OR Degenerative Arthritide OR Degenerative Arthritis OR Osteoarthrosis Deformans OR Arthrosis OR arthrosis OR OA)

#2: TS = (knee OR knees)

#3: TS = ((‘Mesenchymal Stem Cell’ OR ‘Mesenchymal Stem Cells’ OR ‘Mesenchymal Stromal Cell’ OR ‘Mesenchymal Stromal Cells’ OR ‘Mesenchymal Progenitor Cell’ OR ‘Mesenchymal Progenitor Cells’ OR ‘Bone Marrow Stromal Cell’ OR ‘Bone Marrow Stromal Cells’ OR ‘Bone Marrow Stromal Stem Cell’ OR ‘Bone Marrow Stromal Stem Cells’ OR ‘Wharton Jelly Cells’ OR ‘adipose-derived stem cells’ OR ‘Adipose Derived Mesenchymal Stromal Cells’ OR ‘Adipose Tissue-Derived Mesenchymal Stromal Cells’ OR ‘Adipose Tissue Derived Mesenchymal Stromal Cells’ OR ‘Stromal Cells’ OR ‘Stromal Cell’ OR ‘Stem Cells’ OR ‘Stem Cell’ OR ‘MSC’))

#4 TS = ((‘randomized controlled trial’ OR ‘controlled clinical trial’ OR ‘randomized’ OR ‘placebo’ OR ‘randomly’ OR ‘trial’ OR ‘double blind method’ OR ‘double-blind’ OR ‘single blind method’ OR ‘triple blind method’))

#5: #1 AND #2 AND #3 AND #4

## Appendix 2

*Note*: The width of the lines represents the number of studies being compared, and the node size reflects the sample size.

## Appendix 3

*Note*: The funnel plot of network meta-analysis for each outcome indicator.

*Note*: The funnel plot of conventional meta-analysis for each outcome indicator.

## Abbreviations

AD-MSCs: Adipose-derived mesenchymal stem cells
MSCs: Mesenchymal stem cells
BM-MSCs: Bone marrow mesenchymal stem cells
UC-MSCs: Umbilical cord mesenchymal stem cells
KOA: Knee osteoarthritis
RCT: Randomized controlled trials
VAS: Visual Analog Scale
WOMAC: Western Ontario and McMaster Universities Osteoarthritis Index
AEs: Adverse events
SMD: Standardized mean difference
CI: Credible intervals
